# Lay community health worker-led care with mobile decision support for uncontrolled hypertension: a cluster-randomized trial

**DOI:** 10.1038/s41591-026-04208-w

**Published:** 2026-02-12

**Authors:** Felix Gerber, Giuliana Sanchez-Samaniego, Ravi Gupta, Thabo Ishmael Lejone, Thesar Tahirsylaj, Fabian Raeber, Mamakhala Chitja, Malebona Mathulise, Thuso Kabi, Mosoetsi Mokaeane, Malehloa Maphenchane, Manthabiseng Molulela, Makhebe Khomolishoele, Mota Mota, Sesale Masike, Matumaole Bane, Manthati Mofokeng, Mamoronts’ane Pauline Sematle, Retselisitsoe Makabateng, Madavida Mphunyane, Lebohang Sao, Mosa Tlahali, Malitaba Litaba, Dave Brian Basler, Kevin Kindler, Irene Ayakaka, Pauline Grimm, Thilo Burkard, Frédérique Chammartin, Alain Amstutz, Niklaus Daniel Labhardt

**Affiliations:** 1 Division of Clinical Epidemiology, Department of Clinical Research, University Hospital Basel, Basel, Switzerland; 2 University of Basel, Basel, Switzerland; 3 Swiss Tropical and Public Health Institute, Allschwil, Switzerland; 4 SolidarMed Lesotho, Maseru, Lesotho; 5 Ministry of Health Lesotho, Maseru, Lesotho; 6 Faculty of Business, Economics and Informatics, University of Zurich, Zurich, Switzerland; 7 SolidarMed Switzerland, Lucerne, Switzerland; 8 Medical Outpatient Department and Hypertension Centre, ESH Hypertension Centre of Excellence, University Hospital Basel, Basel, Switzerland; 9 Department of Cardiology, University Hospital Basel, Basel, Switzerland; 10 Oslo Center for Biostatistics and Epidemiology, Oslo University Hospital, University of Oslo, Oslo, Norway; 11 Electronic Health Records Group, Population Health Sciences, Bristol Medical School, University of Bristol, Bristol, UK

**Keywords:** Hypertension, Health services, Outcomes research, Health policy

## Abstract

Access to hypertension care remains insufficient, particularly in remote rural areas in resource-limited settings. Community health workers (CHWs), lay providers living in the communities they serve, may help close this gap, but the effectiveness and safety of lay CHW-led hypertension care—including independent initiation and titration of medication—remain uncertain. We conducted a 1:1 cluster-randomized trial nested within the Community-Based Chronic Care Lesotho (ComBaCaL) cohort study in 103 rural villages in Lesotho. Following community-based hypertension screening, 547 nonpregnant adults with blood pressure (BP) ≥140/90 mm Hg were enrolled (274 control and 273 intervention). In intervention clusters, lay CHWs independently prescribed and titrated a fixed-dose combination of amlodipine and hydrochlorothiazide, guided by a mobile clinical decision support system. In control clusters, participants were referred to health facilities for standard care. The primary objective was to assess the effectiveness and safety of lay CHW-led care, with the primary outcome defined as BP <140/90 mm Hg at 12 months. In the intention-to-treat analysis (543 participants with 4 exclusions owing to intercurrent pregnancy), BP control was achieved by 156/271 (58%) versus 130/272 (48%) in intervention and control arms, respectively (adjusted odds ratio 1.52, 95% confidence interval 1.01 to 2.29, *P* = 0.046). A predefined complete case analysis yielded consistent results. No relevant differences in safety outcomes were observed. Among people with uncontrolled hypertension, lay CHW-led, CDSS-supported care was safe and more effective than referral to facility-based professional care. These findings support expanding first-line hypertension management by lay CHWs in remote, resource-limited settings. Clinicaltrials.gov registration: NCT05684055.

## Abstract (web summary)

A cluster-randomized trial in rural Lesotho showed that uncontrolled hypertension diagnosis and management delivered by minimally trained lay persons in the community using a mobile decision support tool was superior to facility referral for improving clinical outcomes.

## Main

Globally, arterial hypertension is the most important risk factor for premature mortality, accounting for 10.8 million or almost 20% of all deaths in 2019^1^. Low- and middle-income countries (LMICs) bear a disproportionate and growing share of the burden, while many high-income countries have managed to reduce the burden of hypertension substantially over the past decades^2^. Besides demographic and lifestyle changes, insufficient access to preventive, diagnostic and therapeutic services are driving the excessively high morbidity and mortality associated with hypertension in LMICs^3^. In 2019, 78% of the 1.3 billion people with hypertension were living in LMICs, with less than 20% of them reaching treatment targets^4^. In LMICs, people living in rural areas, and those with a lower socioeconomic status, face pronounced barriers to accessing hypertension care^5^. Through improved access to antihypertensive medication alone, about 40 million premature deaths could be prevented over the next 25 years^6^.

In Lesotho, a predominantly rural low-income country in southern Africa, a recent population-based survey found a hypertension prevalence of 21.6% among adults aged ≥18 years^7^. Among those affected, 70% were aware of their condition, 67% reported taking treatment, yet only 49% achieved blood pressure (BP) control (<140/90 mm Hg)^8^ and a considerable proportion already showed signs of end-organ damage such as retinopathy and nephropathy^9^. This growing burden of hypertension adds to Lesotho’s high prevalence of human immunodeficiency virus (HIV) and tuberculosis, reflecting the double burden of infectious and noncommunicable diseases^10^. Faced with this challenge and given the constraints of a weak health system and shrinking international funding for chronic care, there is an urgent need for innovative and affordable models of service delivery.

Given the global shortage of healthcare professionals, task-shifting to community health workers (CHWs) has been identified as a promising approach to meet the increasing care needs for hypertension and other chronic conditions in LMICs^11,12^. CHWs are members of the community they serve and receive lower levels of formal education and training than professional healthcare workers^13^. CHWs bring services closer to the community, reduce access barriers such as transport costs, travel time and low awareness, thereby promoting more equitable and less stigmatized access to care^14^. CHW-led hypertension care interventions have been assessed in different settings showing an overall modest effectiveness on BP control^15–17^. Interventions without a pharmacological treatment component were of no or limited effectiveness^15,16,18^, whereas multilevel interventions combining CHW-led screening, monitoring and drug delivery with prescription by physicians have proven highly effective in several large-scale trials^17,19–22^. However, reliance on physician prescriptions may limit the scalability and workload redistribution of such interventions.

Only one trial conducted in rural China evaluated an intervention with independent drug prescription, titration and monitoring by CHWs^23,24^. The intervention significantly improved BP control rates and reduced cardiovascular events and mortality compared to enhanced usual care^23,24^. However, CHWs in China (‘village doctors’) have a substantially higher level of medical education than CHWs in most other countries, including Lesotho, and it remains unclear whether the findings can be replicated in systems outside China with lay CHWs who do not have a formal medical education or clinical experience^25^. Digital clinical decision support systems (CDSS) and simplified treatment protocols using fixed-dose combination pills may enable lay CHWs to provide hypertension care safely and effectively^26,27^. CDSS directly assist clinical decision-making by providing personalized assessments and recommendations on the basis of individual patient characteristics^28^. By offering algorithmic guidance and facilitating remote supervision, mobile CDSS may enhance the quality, efficiency and scope of CHW services^29^.

Lesotho has an established community health system in which lay persons, elected by their communities and accredited after a 6-week training, serve part-time as CHWs and receive a stipend. Reflecting the country’s mountainous geography with many small, remote villages, the national strategy targets a CHW-to-population ratio of 1:250 (ref. ^30^). The responsibilities of Lesotho’s lay CHWs include health promotion and education, nutrition and growth monitoring, and the sensitization, screening and referral of individuals for communicable diseases such as HIV and tuberculosis. As in most countries in the region, hypertension diagnosis and treatment are not yet part of CHWs’ routine tasks in Lesotho.

This cluster-randomized trial aimed to assess the safety and effectiveness of an intervention including independent prescription, monitoring and titration of antihypertensive medication by CDSS-assisted lay CHWs among people with uncontrolled hypertension in rural Lesotho. It addresses several of the top research priorities for improving hypertension care identified by the World Health Organization (WHO), including the development and evaluation of systems for hypertension care delivery closer to home, CHW-led medication prescription and titration, a digital approach to improving retention in care, and task-sharing that addresses barriers to accessibility^31^. The intervention was developed in collaboration with local community members and the Lesotho Ministry of Health on the basis of a local noncommunicable diseases prevalence survey and burden assessment^7,8^, a scoping literature review^32^ and a pilot study in the local setting^33^. The trial was conducted within the Community-Based Chronic Disease Care Lesotho (ComBaCaL) cohort study (NCT05596773)^34,35^. This cohort comprises the population of 103 villages in rural Lesotho and serves as platform for the investigation of chronic diseases and their management^36,37^. It is embedded within the government-led community health program and managed by local lay CHWs.

## Results

### Participant enrollment and baseline characteristics

**Fig. 1:**
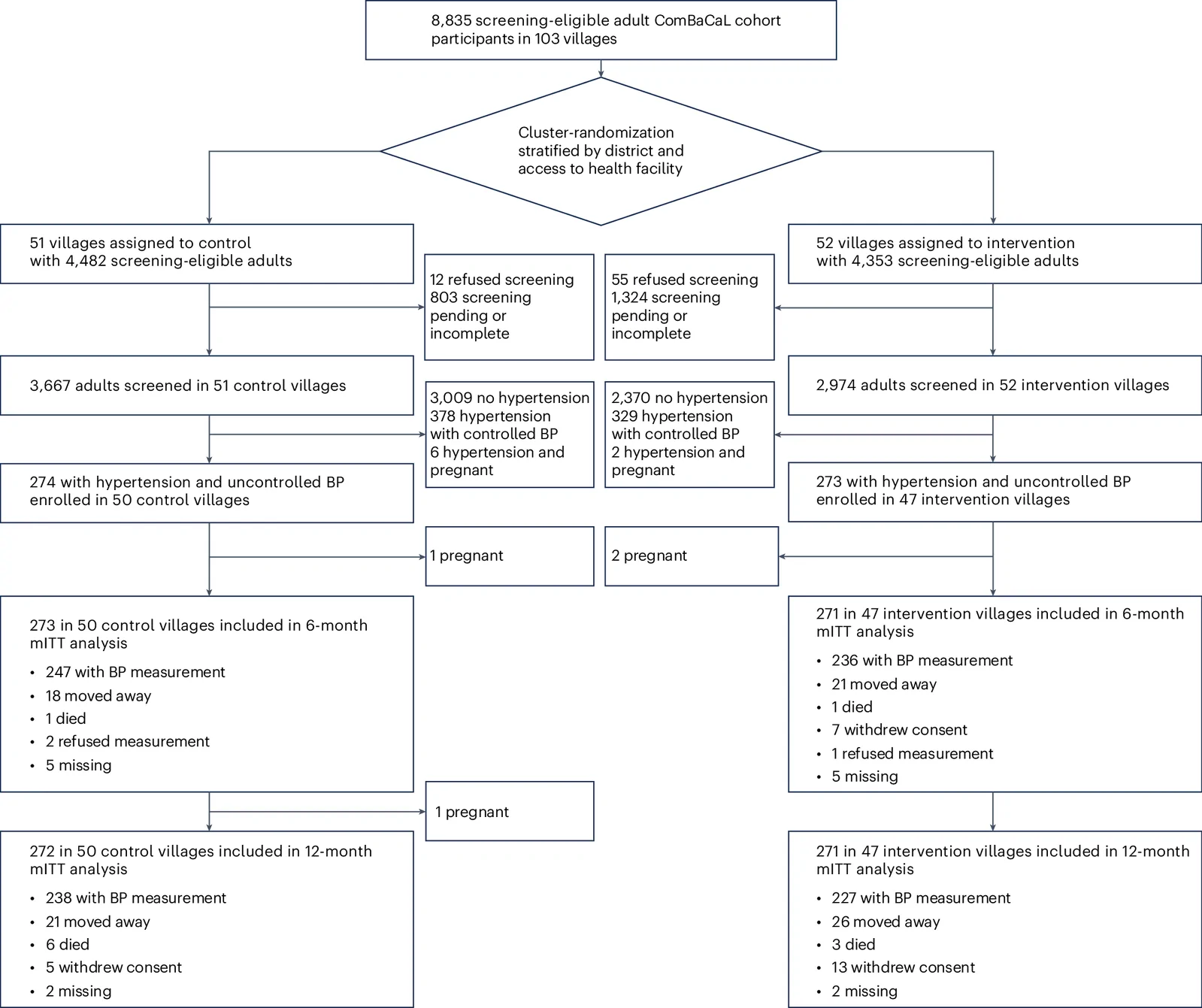
Participant flow chart. CONSORT flow diagram of clusters and participants. mITT, modified ITT.

All 103 villages in the ComBaCaL cohort were included and randomized; no clusters declined participation or were excluded. Between 8 September 2023 and 31 January 2024, lay CHWs screened 6,641 out of 8,835 eligible adult ComBaCaL cohort participants for hypertension during home visits across 103 rural villages. They identified 1,262 cohort participants with hypertension, out of which 547 (274 control and 273 intervention) from 97 villages (47 intervention and 50 control) had uncontrolled BP levels (≥140/90 mm Hg) and were not pregnant (Fig. 1). In six villages, no cohort participants were eligible for this trial.

The trial participants had a mean age of 62.3 years and a mean BP of 149.0 (s.d. 16.8) over 95.0 (s.d. 10.1) mm Hg, 75.0% were female, 44.6% reported use of antihypertensive medication at enrollment, 35.1% were smokers, 8.4% had diabetes, 15.7% reported living with HIV, 1.3% had heart failure and 4.9% had a history of stroke or myocardial infarction. No substantial imbalance across the arms was observed (Table 1 and Supplementary Table 1).

**Table 1:** Baseline characteristics of participants by study arm

Cluster-level characteristics	Control	Intervention	Total
Villages, *n*	50	47	97
Adult cohort participants eligible for screening per village, median (IQR)	85.0 (67.8–101.3)	78.0 (67.5–99.0)	81.0 (67.5–101.0)
Participants per village, median (IQR)	6.0 (3.0–7.0)	5.0 (3.0–8.5)	5.0 (3.0–8.0)
Range	1–15	1–17	1–17
Hard access to health facility^a^, *n* (%)	27 (54.0)	26 (55.3)	53 (54.6)
District Butha-Buthe (versus Mokhotlong), *n* (%)	27 (54.0)	24 (51.1)	51 (52.6)
**Individual-level characteristics**			
Participants, *n*	274	273	547
**Demographics and anthropometrics**			
Female, *n* (%)	200 (73.0)	210 (76.9)	410 (75.0)
Age (years), mean (s.d.)	61.6 (17.4)	63.0 (16.2)	62.3 (16.8)
BMI (kg m^−^^2^), mean (s.d.)	27.0 (6.7)	28.5 (6.6)	27.8 (6.7)
Abdominal circumference (cm), mean (s.d.)	90.8 (14.3)	90.6 (15.5)	90.7 (14.9)
Education at secondary school or higher, *n* (%)	57 (20.8)	42 (15.4)	99 (18.1)
Working for pay or self-employed, *n* (%)	99 (36.1%)	117 (42.9%)	216 (39.5%)
Married or in a stable relationship, *n* (%)	143 (52.2)	143 (52.4)	286 (52.3)
**BP**			
Systolic BP (mm Hg), mean (s.d.)	147.5 (17.0)	150.5 (16.5)	149.0 (16.8)
Diastolic BP (mm Hg), mean (s.d.)	94.5 (9.3)	95.4 (10.9)	95.0 (10.1)
Engaged in hypertension care, *n* (%)	123 (44.9)	121 (44.3)	244 (44.6)
**Cardiovascular disease risk, lifestyle and comorbidities**			
Cardiovascular disease risk ≥10%^b^, *n* (%)	131 (50.4)	150 (61.2)	281 (55.6)
Current smoking, *n* (%)	86 (31.4)	106 (38.8)	192 (35.1)
Alcohol consumption ≥1 day per week, *n* (%)	63 (23.1)	62 (22.8)	125 (22.9)
Moderate or high physical activity^c^, *n* (%)	217 (79.2)	231 (85.6)	448 (82.4)
In care for a chronic condition^d^, *n* (%)	52 (19.0)	68 (24.9)	120 (21.9)
Heart failure, *n* (%)	4 (1.5)	3 (1.1)	7 (1.3)
History of stroke or myocardial infarction (%)	14 (5.1)	13 (4.8)	27 (4.9)
Diabetes, *n* (%)	23 (8.4)	23 (8.4)	46 (8.4)
HIV, *n* (%)	39 (14.2)	47 (17.2)	86 (15.7)
Statin use among eligible participants^e^, *n* (%)	0 (0.0)	9 (5.7)	9 (3.1)
Total cholesterol (mg dl^−1^), mean (s.d.)	153.2 (44.6)	154.9 (40.6)	154.0 (41.6)
Always or often adding salt to food while eating^f^, *n* (%)	45 (16.4)	44 (16.2)	89 (16.3)

^a^Needing to cross a mountain or river or travel >10 km to the nearest health facility.

^b^Ten-year risk for a cardiovascular event, using the WHO laboratory-based cardiovascular risk calculator.

^c^Self-reported physical activity using the International Physical Activity Questionnaire Short Form (IPAQ-S).

^d^HIV and/or diabetes.

^e^Participants with a history of stroke, myocardial infarction, diabetes or chronic kidney disease or aged ≥50 years and having a BMI of ≥30 kg m^−^^2^ or aged ≥50 years and currently smoking were eligible for statin. Number of participants eligible was 289 (129 in control and 159 in intervention).

^f^Assessed using the WHO STEPS instrument.

Missing data: BMI: 3 (control), 3 (intervention); abdominal circumference: 2, 8; education: 0, 1; use of antihypertensive medication: 0, 1; WHO cardiovascular risk: 14, 28; alcohol consumption: 1, 1; physical activity: 0, 3; total cholesterol: 14, 28; adding salt to food: 0, 1.

### Primary outcome

For the primary outcome of BP control (<140/90 mm Hg) at 12 months, the intention-to-treat (ITT) analysis included 543 participants (4 were excluded owing to pregnancy during follow-up). BP control was achieved by 156 of 271 participants (58%) in the intervention arm and 130 of 272 participants (48%) in the control arm (Table 2). The adjusted odds ratio (aOR) of the intervention on controlled BP was aOR 1.52 (95% confidence interval [CI] 1.01 to 2.29, *P* = 0.046). This corresponds to an average intervention effect of 12.5% (95% CI 2.2 to 22.5%) increase in BP control rate. Diagnostic checks indicated no major violations of model assumptions; random effects were approximately normally distributed, and the relationship of age with the outcome was linear on the logit scale.

**Table 2:** Intervention effects on primary and selected secondary and prespecified exploratory endpoints. Effects on all other secondary endpoints are available in Extended Data Table 2

	Control	Intervention	aOR or adjusted mean difference (95% CIs)^a^
**Primary endpoint analysis**^b,o^	*N* = 272	*N* = 271	
BP <140/90 mm Hg at 12 months, *n* (%), ITT^c^	130 (47.8)	156 (57.6)	1.52 (1.01 to 2.29)
**Sensitivity analysis**^d^^,p^	*N* = 238	*N* = 227	
BP <140/90 mm Hg at 12 months, *n* (%), complete case	130 (54.6)	156 (68.7)	1.95 (1.23 to 3.10)
**Secondary endpoints at 12** **months**^b^	*N* = 272	*N* = 271	
SBP, mm Hg, mean (s.d.)^q^	134.7 (17.6)	131.1 (16.0)	−4.2 (−7.6 to −0.8)
DBP, mm Hg, mean (s.d.)^q^	86.2 (10.8)	84.1 (9.9)	−2.4 (−4.6 to −0.2)
Engagement in care^e^, *n* (%), ITT^f^	196 (72.1)	220 (81.2)	1.65 (1.09 to 2.51)
Engagement in care^f^, *n* (%), complete case^g^	196 (82.0)	220 (96.9)	12.51 (3.45 to 45.34)
Linkage to care^h^, *n* (%)	56 (37.3)	133 (88.1)	13.20 (6.75 to 25.83)
Already on treatment at baseline	122	120	
Adherence to antihypertensive medication^i^, *n* (%)	135 (87.7)	148 (77.9)	0.49 (0.18 to 1.29)
Not on treatment at 12 months	115	80	
Cardiovascular disease risk ≥10%^j^, *n* (%)	118 (49.8)	125 (55.1)	0.94 (0.45 to 1.96)
Total cholesterol, mg dl^−1^, mean (s.d.)	162.2 (44.1)	163.0 (55.7)	−0.9 (−11.4 to 9.6)
Current smoking, *n* (%)	89 (37.2)	105 (46.3)	1.60 (0.59 to 4.31)
Statin use among eligible participants^k^, *n* (%)	5 (5.0)	37 (27.6)	1.28 (0.8 to 2.03)
Not eligible for statin use	137	99	
**Secondary endpoints at 6** **months**^l^	*N* = 273	*N* = 271	
BP <140/90 mm Hg, *n* (%), ITT^c,s^	120 (44.0)	144 (53.1)	1.37 (0.91 to 2.05)
BP <140/90 mm Hg, *n* (%), complete case^m,t^	120 (48.6)	144 (61.0)	1.64 (1.08 to 2.49)
SBP, mm Hg, mean (s.d.)^r^	135.6 (17.4)	132.7 (15.6)	−3.6 (−6.5 to −0.6)
DBP, mm Hg, mean (s.d.)^r^	87.0 (11.3)	84.7 (9.5)	−2.6 (−4.4 to −0.8)
Engagement in care^e^, *n* (%), ITT^f^	190 (69.6)	220 (81.2)	1.94 (1.27 to 2.95)
Engagement in care^e^, *n* (%), complete case^n^	190 (76.0)	220 (92.4)	4.97 (2.20 to 11.25)
Linkage to care^h^, *n* (%)	44 (29.3)	130 (86.1)	16.65 (8.30 to 33.41)
Already in care at baseline	123	120	
Adherence to antihypertensive medication^i^, *n* (%)	124 (81.0)	139 (72.8)	0.56 (0.23 to 1.38)
Not on treatment	121	77	
Cardiovascular risk ≥10%^j^, *n* (%)	117 (49.0)	115 (52.8)	0.79 (0.38 to 1.68)
Total cholesterol, mg dl^−1^, mean (s.d.)	153.5 (38.9)	156.2 (45.9)	−1.3 (−10.2 to 7.6)
Current smoking, *n* (%)	85 (34.1)	94 (39.7)	0.73 (0.27 to 1.99)
Statin use among eligible participants^k^, *n* (%)	4 (3.7)	59 (36.0)	2.96 (1.44 to 6.05)
Not eligible for statin use	143	105	

^a^Effects are derived from mixed-effect regression models adjusted for clustering and covariates (sex, age, district, facility access and baseline values when appropriate).

^b^Four participants excluded owing to pregnancy between enrollment and 12 months.

^c^Participants with missing BP measurement were considered as having a BP ≥140/90 mm Hg.

^d^Only including participants with available BP measurement at 12 months: 238 control and 227 intervention.

^e^Reporting current use of antihypertensive medication or reaching treatment target without medication.

^f^Participants with missing measurement were considered as not being engaged in care.

^g^Only including participants with available endpoint data at 12 months: 240 control and 227 intervention.

^h^Proportion of participants who have initiated antihypertensive treatment since enrollment among those not taking treatment at baseline.

^i^Reporting no dose of antihypertensive medication missed over the last 4 days.

^j^Ten-year risk for a cardiovascular event, using the WHO laboratory-based cardiovascular risk calculator.

^k^Predefined exploratory endpoint. Statin eligible: participants with history of stroke, myocardial infarction, diabetes or chronic kidney disease or aged ≥50 years and having a BMI of ≥30 kg m^−^^2^ or aged ≥50 years and currently smoking.

^l^Three participants excluded owing to pregnancy between enrollment and 6 months.

^m^Only including participants with available BP measurement at 6 months: 247 control and 236 intervention.

^n^Only including those with available endpoint data at 6 months: 250 control and 236 intervention.

^o^Intracluster correlation coefficient (ICC) of 0.06.

^p^ICC of 0.07.

^q^ICC SBP of 0.06 and ICC DBP of 0.11.

^r^ICC SBP of 0.01 and ICC DBP of 0.02.

^s^ICC of 0.06.

^t^ICC of 0.05.

Missing data: SBP and DBP at 12 months: 34 control and 44 intervention. SBP and DBP at 6 months: 26 control and 35 intervention.

In a predefined sensitivity analysis only including participants with available BP measurements at 12 months (complete case), BP control rates were 156/227 (69%) and 130/238 (55%) in the intervention and control arm, respectively, with an aOR of 1.95 (1.23 to 3.10).

**Extended Data Fig. 1:**
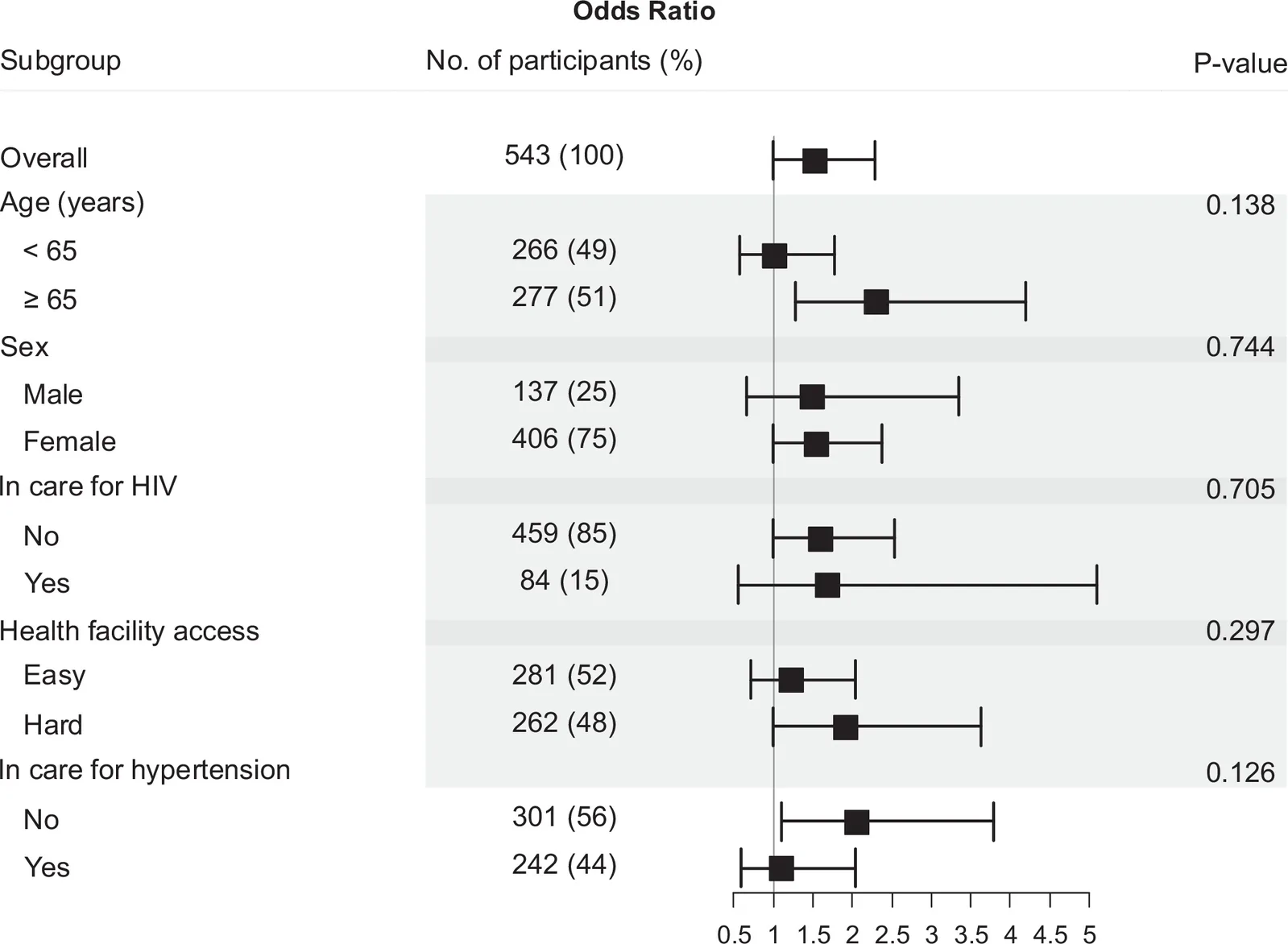
Predefined subgroup analyses for blood pressure control rates ( < 140/90 mmHg) at 12 months in intervention versus control. Adjusted odds ratio and its 95% CI were obtained by mixed-effect regression models adjusted for clustering and covariates (sex, age, district, facility access and baseline values when appropriate). The p value was two-sided and was not adjusted for multiple comparison. Hard health facility access defined as needing to cross a mountain or river or travel >10 km to the nearest health facility.

Interaction *P* values for the prespecified potential effect modifiers (sex, age, engagement in hypertension care at baseline, access to health facility and engagement in HIV care) were larger than 0.1 and therefore no subgroup credibility assessments were performed (Extended Data Table 1 and Extended Data Fig. 1).

### Secondary outcomes

At 6 months, the BP control rates in the ITT analysis were 144/271 (53%) in the intervention arm and 120/273 (44%) in the control arm, with an aOR of 1.37 (0.91 to 2.05). The mean systolic BP (SBP) and diastolic BP (DBP) after 12 months were −4.2 mm Hg (−7.6 to −0.8 mm Hg) and −2.4 mm Hg (−4.6 to −0.2 mm Hg) lower in the intervention compared to the control arm.

**Fig. 2:**
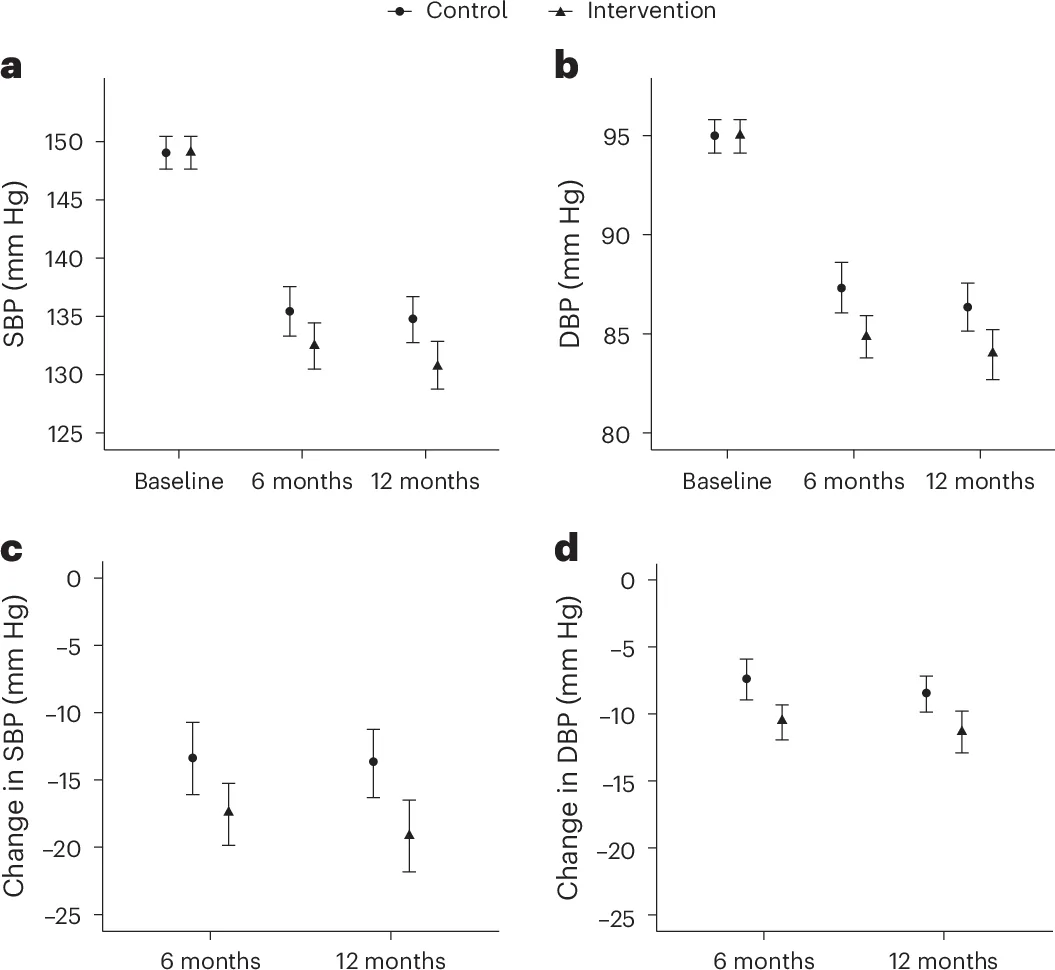
Mean BP in intervention and control arms. **a**–**d**, Standardized mean SBP (**a**), mean DBP (**b**), change in SBP (**c**) and change in DBP (**d**) in control and intervention arms. Statistics are derived using marginal standardization. Error bars reflect the 2.5–97.5th percentiles from 1,000 bootstrap samples.

Compared to baseline, the mean SBP decreased by −19.2 mm Hg (−21.8 to −16.5 mm Hg) in the intervention and by −13.7 mm Hg (−16.3 to −11.2 mm Hg) in the control arm after 12 months. The mean DBP at 12 months decreased by −11.4 mm Hg (−12.9 to −9.8 mm Hg) in the intervention and by −8.5 mm Hg (−9.9 to −7.2 mm Hg) in the control arm (Fig. 2 and Table 2). At 6 months, the mean SBP decreased by −17.1 mm Hg (−19.5 to −14.8 mm Hg) in the intervention and by −13.0 mm Hg (−15.6 to −10.4 mm Hg) in the control arm. The mean DBP at 6 months decreased by −10.5 mm Hg (−11.9 to −9.3 mm Hg) in the intervention and by −7.4 mm Hg (−8.8 to −5.9 mm Hg) in the control arm.

**Fig. 3:**
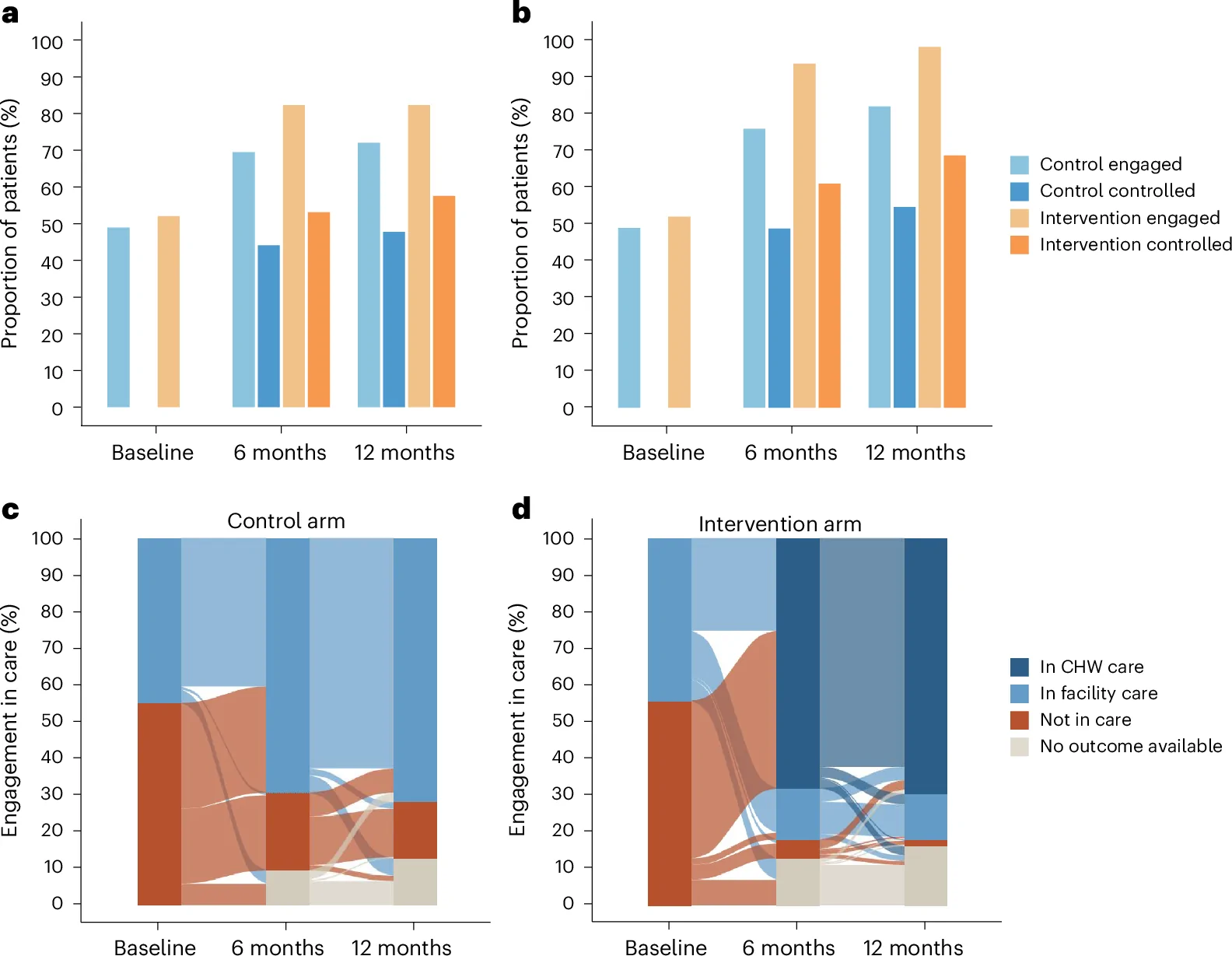
Engagement in care, BP control rates and type of care provider. **a**,**b**, Observed proportion of participants engaged in care and reaching the control target of <140/90 mm Hg at baseline, 6 months and 12 months by study arm: ITT population (*n* = 547 at baseline, *n* = 544 at 6 months and *n* = 543 at 12 months) with participants with missing endpoint data considered as not engaged and not controlled (**a**) and the complete-case population (*n* = 547 at baseline, *n* = 483 at 6 months and *n* = 465 at 12 months) only considering participants with available endpoint data (**b**). **c**,**d**, Engagement in care at baseline, 6 months and 12 months by study arm and provider: control arm (**c**) and intervention arm (**d**).

At 6 months, 220/271 (81.2%) participants in the intervention arm and 190/273 (69.6%) in the control arm were engaged in care, with an aOR of 1.94 (1.27 to 2.95) (Table 2 and Fig. 3a,b). Among those not engaged in hypertension care at baseline, in the intervention arm 130/151 (86.1%) participants linked to care by 6 months compared to 44/150 (29.3%) in the control arm, with an aOR of 16.65 (8.30 to 33.41) (Table 3).

**Table 3:** Safety set analysis and safety endpoints

	Control	Intervention	aOR (95% CIs)
**Safety set analysis at 12** **months**^a^	*N* = 206	*N* = 258	
BP <140/90 mm Hg, *n* (%)^b^	94 (45.6)	156 (60.5)	1.93 (1.25 to 2.97)
**Safety set analysis at 6** **months**^a^	*N* = 215	*N* = 255	
BP <140/90 mm Hg, *n* (%)^b^	86 (40.0)	144 (56.5)	1.94 (1.27 to 2.96)
**Safety endpoints at 12** **months**	*N* = 274	*N* = 273	
Deaths, *n* (%)	6 (2.2)	3 (1.1)	
Nonfatal SAE^c^, *n* (%)	2 (0.7)	0 (0.0)	
AESI^d^, *n* (%)	1 (0.4)	9 (3.3)	
Stroke, *n* (%)	1 (0.4)	0 (0.0)	
Side effects of antihypertensive medication, *n* (%)	0 (0.0)	9 (3.3)	

^a^Only considering outcomes of participants with at least one visit for hypertension care by the CHW in the intervention arm or at the health facility in the control arm.

^b^Participants with missing BP measurement were considered as having a BP ≥140/90 mm Hg.

^c^SAEs defined as death, hospitalization, life-threatening event or event leading to persistent disability.

^d^Adverse events of special interest defined as adverse events consistent with hypertension complications, such as myocardial infarction, stroke, heart failure or chronic kidney disease as well as adverse events related to the intake of antihypertensive medication leading to the discontinuation of the medication concerned.

At 12 months, 220/271 (81.2%) participants were engaged in care in the intervention arm versus 196/272 (72.1%) in the control arm, with an aOR of 1.65 (1.09 to 2.51).

There were no significant differences between arms in the estimated 10-year cardiovascular event risk, body weight, body mass index (BMI), abdominal circumference, smoking, alcohol consumption, adherence to antihypertensive medication and dietary habits (Table 2, Extended Data Table 2 and Supplementary Tables 2 and 3). The proportion of participants self-reportedly engaging in moderate or high physical activity was higher in the intervention arm at 12 months but not at 6 months (Extended Data Table 2).

### Exploratory outcomes

At 6 months, among the 220 participants engaged in care in the intervention arm, 186/220 (84.5%) were in care with their lay CHW and 34/220 (15.5%) at the health facility (2 owing to intolerance to amlodipine or hydrochlorothiazide, 9 did not reach the treatment target with amlodipine and hydrochlorothiazide, and 23 preferred facility-based care). At 12 months, among the 220 participants engaged in care in the intervention arm, 190/220 (86.4%) were in care with their lay CHW and 30/220 (13.6%) at the health facility (3 because of intolerance to amlodipine or hydrochlorothiazide, 12 did not reach the treatment targets with amlodipine and hydrochlorothiazide, and 15 preferred facility-based care) (Fig. 3c,d). The proportion of participants using statins among those eligible was higher in the intervention compared to the control arm at 6 months but not at 12 months (Table 2).

### Safety outcomes

By 12 months, six participants in the control arm and three in the intervention arm had died. Two participants in the control arm have been hospitalized, while no nonfatal serious adverse events (SAEs) were reported in the intervention group. None of the events were related to study participation. One adverse event of special interest was observed the control group and nine in the intervention group. All adverse events of special interest in the intervention group were related to ankle swelling in association with intake of amlodipine and resolved after switching antihypertensive medication. One stroke event was reported in the control arm.

In a safety set analysis—considering only outcomes of participants with at least one hypertension care visit by the CHW in the intervention arm or at the health facility in the control arm—BP control rates at 12 months were 156/258 (60.5%) in the intervention arm and 94/206 (45.6%) in the control arm (aOR 1.93 [1.25 to 2.97]), with similar results observed at 6 months (Table 3).

## Discussion

In this cluster-randomized trial in rural Lesotho, lay CHWs who independently initiated, dispensed and titrated antihypertensive medication with the support of a CDSS achieved substantially higher rates of BP control for uncontrolled hypertension at 12 months than referral to professional, facility-based care after community-based screening. The intervention also improved engagement in care and linkage to treatment, without safety concerns. These findings highlight the potential of task-shifting models involving minimally trained CHWs in similar low-income settings.

Given the increasing number of people requiring hypertension care and the limited professional healthcare workforce in LMICs, the need for care models that include task-shifting to nonphysician healthcare workers is evident and recommended by the WHO^12^. Interventions combining CHW-led screening, counseling, drug delivery and monitoring services with physician prescription were found to significantly reduce BP levels and to be cost-effective in several large-scale trials^19–22^. The HOPE-4 study, conducted in Colombia and Malaysia, showed a significant cardiovascular risk and BP reduction through nonphysician healthcare worker-led cardiovascular risk screening using a CDSS application, algorithm-guided treatment recommendations, lifestyle counseling and drug delivery after physician prescription^19^. HOPE-4 intervention led to a large mean SBP reduction of 11.5 mm Hg (ref. ^19^). However, the real-world feasibility of the findings may be limited because the intervention was not fully embedded within the existing health system infrastructure with nonphysician health workers being employed for the study and medication being provided free of charge only in the intervention arm^38^.

The China Rural Hypertension Control Project (CRHCP) was the first study that demonstrated the feasibility and effectiveness of a multifaceted intervention that included independent drug prescription, monitoring and titration by CHWs on BP and mortality reduction^23,24^. Enrolling 33,995 individuals with uncontrolled BP in rural China, the intervention achieved an average SBP reduction of 14.5 mm Hg. However, these findings may not be generalizable to other LMICs^25^. CHWs in China, called ‘village doctors’, are accredited after 3 years of medical education or 20 years of continuous practicing in village clinics and have routine prescription rights for essential medications, unlike CHWs in most LMICs who do not receive a formal medical education and usually only a stipend instead of a full salary^39^. Further, in the CRHCP, participants were provided with automated BP machines and trained on home self-measurements^40^, a strategy that may not be feasible and cost-effective in most LMIC settings^25^. Health system components of the intervention, such as provision of discounted or free medications and the use of hypertension control rates as a metric for resource allocation at health system level, may be challenging to implement in other settings.

The intervention in our study was designed to address some of the limitations identified in previous landmark studies. As opposed to the CRHCP trial conducted in China, in our trial, CHWs were lay people who received only a 1-week training on data collection, screening and diagnosis of hypertension with an additional 2 days of training on the management of hypertension. The trial addresses several of the top research priorities for improving hypertension care identified by the WHO and responds to calls for contextualized clinical and health system hypertension research in Africa^31,41^. The intervention was integrated within the routine government CHW system. Participating CHWs were lay people elected by the village population according to the national Village Health Program Policy^42^. They reported to the supervising health facility during routine monthly meetings, where they also received medication supply through the routine supply chain system. The study team only assisted in remote monitoring through the CDSS application to ensure participant safety and completeness of data. The CDSS application that guided CHWs in service delivery and data collection through algorithmic guidance including algorithmic task scheduling was on the basis of the widely available open source, offline-first, interoperable Community Health Toolkit software platform^43^. Screening, diagnosis and treatment algorithms applied by CHWs followed national guidelines with no formal intervention at the facility level. The intervention may thus be easily adoptable and scalable in different settings, especially where a CHW system is already established.

Our intervention contained a cardiovascular risk assessment along with CHW-led statin prescriptions. Unlike the HOPE-4 study, we did not observe an effect on the estimated 10-year cardiovascular risk. This discrepancy may be due to the more stringent eligibility criteria for statin prescriptions (59% of participants eligible compared to 93% in HOPE-4), the use of different cardiovascular risk assessment tools (WHO risk calculator versus Framingham risk score) and the insufficient uptake of statins in our study to which difficulties with supply and the CDSS application may have contributed. Our findings, alongside those from the HOPE-4 study, demonstrate the feasibility of algorithm-guided cardiovascular risk assessments conducted by CHWs. The use of fixed-dose combinations that include statins and antihypertensive drugs might be an enabling factor to increase statin uptake and adherence in future CHW-led interventions^44^. Like previous studies, we did not observe relevant effects on behavioral cardiovascular risk factors and associated outcomes, such as BMI or abdominal circumference^21,45–47^. Further research is required on how the effectiveness of CHW-led lifestyle counseling in hypertension management could be improved^48^.

Beyond its clinical effectiveness, the intervention demonstrated real-world feasibility: lay CHWs independently delivered guideline-based hypertension care at the village level, thereby reducing reliance on health facilities and expanding access to treatment in remote rural areas. A previous study within the ComBaCaL cohort demonstrated that CHW-led screening and diagnosis of hypertension and diabetes achieved high population coverage at moderate cost^49^. The whole ComBaCaL project and its studies were co-developed with the Lesotho Ministry of Health, with active engagement of community leaders and CHWs, which strengthened feasibility, cultural appropriateness and alignment with national health priorities. These features enhance the relevance of our findings and support their potential scalability in similar low-income rural settings.

However, this study has several limitations. First, it only included participants with uncontrolled BP at baseline. To assess the effectiveness of the intervention among all people living with hypertension, another trial within the ComBaCaL cohort was conducted that enrolled participants with controlled BP at baseline^36^. Second, while the CHWs in our study were part of the government CHW system, they received an incentive of roughly US$20 per month in addition to the regular government stipend of about US$40 to compensate the study-related workload. All CHWs in both arms received the same nonperformance-based stipend, which compensated study-related workload and cannot explain between-group differences. Sustainable scale-up will require realistic workload, adequate support structures and cost-effectiveness assessments to inform appropriate CHW compensation. Third, the CHWs who delivered the intervention also assessed all endpoints. Desirability and recall bias may affect the self-reported behavioral outcomes. The CHWs were trained and guided by the CDSS application for correct BP measurements with the application automatically calculating the average of the last two out of three entered measurements as outcome. We thus deem the risk of measurement bias on the primary outcome as small. Fourth, we used a pragmatic titration algorithm that only included a combination of amlodipine and hydrochlorothiazide at two dosages and the same treatment target of <140/90 mm Hg for all participants. Amlodipine and hydrochlorothiazide are the only drugs available as a fixed-dose combination pill at an affordable price. They have a favorable effectiveness and safety profile, especially in the absence of electrolyte monitoring^41,50^. However, this pragmatic approach led to suboptimal treatment for participants with comorbidities, such as diabetes or chronic kidney disease, which would benefit from a renin–angiotensin–aldosterone-system blocker and younger participants for whom more stringent treatment targets may be recommended. On the basis of our experience, increasing the treatment complexity for lay CHW-led, CDSS-supported management may be considered in future projects. Fifth, this study assessed the effectiveness of a lay CHW-led hypertension and cardiovascular risk management, while there is a consensus that in most settings, CHWs should provide a comprehensive, integrated service package rather than single-disease focused interventions. However, this trial was implemented in parallel with a similar trial within the ComBaCaL cohort assessing the effectiveness of lay CHW-led diabetes management^37,51^. Further, the CHWs conducted routine government tasks related to health promotion and linkage services that were not formally monitored within the study. Thus, the intervention was partially integrated with other services, while a formal assessment of the integrated care delivery is lacking. Further research is needed to assess both the (cost)effectiveness of targeted interventions for other conditions and their integration to develop an evidence-based CHW-led essential service package. The findings of our trial are likely to be generalizable to other rural low-income settings where a lay CHW system is already in place or being rolled out. However, generalizability may be more limited in urban contexts or in health systems with different task-shifting regulations.

This cluster-randomized trial demonstrates that lay CHW-led hypertension care, supported by a mobile CDSS, improved BP control at 12 months without safety concerns. Health policies should consider empowering lay CHWs to independently initiate and titrate antihypertensive treatment for uncomplicated hypertension to address prevailing gaps in hypertension care.

## Methods

### Study design and setting

We conducted a 1:1 cluster-randomized, open-label, superiority trial nested within the ComBaCaL cohort study following the Trials within Cohorts (TwiCs) design^52^. The ComBaCaL cohort study was conducted in 103 randomly selected villages in the two rural districts, Butha-Buthe and Mokhothlong, in Lesotho, a small, landlocked country surrounded by South Africa^34^. In each ComBaCaL village, a lay person was elected by the community according to the procedures outlined in the Lesotho Ministry of Health Village Health Program policy^42^. Lay CHWs play an important role in the Lesotho healthcare system providing health promotion services and linking the community to facility-based care, mainly for maternal and child health and HIV^42,53,54^. Lesotho is a typical example of an African LMIC where a developing health system is facing the double burden of still highly prevalent infectious diseases (HIV/AIDS and tuberculosis) in combination with a rapidly growing noncommunicable disease epidemic^7,55,56^. The trial was designed and implemented in partnership with the Lesotho Ministry of Health, who acted as co-investigators, and with input from community leaders in participating villages. Local CHWs were centrally involved in planning and delivery, ensuring feasibility and cultural appropriateness.

### Screening and enrollment

Participants were recruited among the ComBaCaL cohort population, which included all consenting inhabitants of the 103 randomly selected rural villages. ComBaCaL cohort participants aged 18 years or older were screened for hypertension by their lay CHW during home visits according to the diagnostic algorithm of the Lesotho Standard Treatment Guidelines^57^ (Supplementary Fig. 1). All nonpregnant adult ComBaCaL cohort participants with hypertension, defined as reporting an intake of antihypertensive medication or being newly diagnosed during screening with uncontrolled BP levels (≥140/90 mm Hg) were eligible. At enrollment, lay CHWs collected baseline sociodemographic and clinical information, including self-reported sex, age, medical history and BP. BP was measured using automated devices (Omron M3 Comfort [HEM7131-E]). Measurements were taken after selecting the appropriate cuff size, with participants seated comfortably—feet flat on the floor, arm supported and remaining still and silent for 5 min. During the first visit, BP was measured in both arms to determine the reference arm for subsequent assessments. The arm with higher systolic BP was identified as reference arm and used for all subsequent BP measurements. The BP value was calculated as the mean of the last two out of three consecutive measurements at intervals of 1 min. For the diagnosis of hypertension, two elevated measurements in the range of 140–179/90–109 mm Hg on two different days or two measurements of 180/110 mm Hg or higher on the same day, at least 30 min apart, were required.

### Randomization and blinding

Half of the ComBaCaL cohort villages were randomly allocated to the intervention group by a statistician not involved in the study before the start of the hypertension screening. The randomization was stratified by district (Butha-Buthe versus Mokhothlong) and access to health facilities (easy versus difficult access, defined as needing to cross a mountain or river or travel >10 km to the nearest health facility). Participants were blinded to the allocation, meaning that participants in the control villages were not aware of the intervention being implemented in other villages. The lay CHWs who enrolled participants, provided the intervention and collected endpoint data were not blinded.

### Procedures

In each village, there was one lay CHW, equipped with a password-protected tablet loaded with a tailored CDSS application. The CDSS application provided algorithmic guidance for all study procedures and served as a data collection tool. The application was configured by the study team on the basis of the open source Community Health Toolkit (CHT) Core Framework (versions 3.12, 3.14, 4.6 and 4.13), a widely used, open source software toolkit designed for community health systems^58^. As a progressive web application with an offline-first approach, CHT is particularly well-suited for settings with limited internet connectivity. It provides a core framework and configuration code that enable the rapid development and deployment of scalable digital health applications on Android-based mobile devices^58^. The application allowed the lay CHWs to register household and clients, document electronic written consent and guided them via automated task scheduling and structured questionnaires through all clinical procedures and data collection. Furthermore, the application provided individual client overviews and an aggregated data overview with target metrics. Data were synchronized regularly to a secure server hosted at the University Hospital Basel, Switzerland. Data were monitored locally by the CHW supervisors and centrally by the data management team of the University Hospital Basel.

The lay CHWs were elected by the village population in a participatory process according to the Lesotho Ministry of Health Village Health Program policy^42^. All lay CHWs received a 1-week training on data collection, screening and diagnosis of hypertension. Lay CHWs in the intervention villages received an additional 2 days of training on the intervention services. In both control and intervention villages, the lay CHWs received the regular government stipend of roughly US$40 per month and an additional US$20 to compensate the work related to study participation.

In control villages, lay CHWs offered lifestyle counseling and referred participants to their closest health facility for further care after diagnosis and enrollment. At 6 months, they conducted a check-up with lifestyle counseling and another referral in case the participant had not yet linked to care.

In intervention villages, lay CHWs offered a community-based hypertension care package that included lifestyle counseling, prescription of antihypertensive fixed-dose combinations containing amlodipine and hydrochlorothiazide at low dose (5 mg/12.5 mg) or high dose (10 mg/25 mg), as well as lipid-lowering (atorvastatin 10 mg) and antiplatelet (acetyl salicylic acid 100 mg) treatment for eligible participants (Supplementary Fig. 2). Screening, diagnosis, as well as treatment initiation and monitoring followed the recommendations of the current Lesotho Standard Treatment Guidelines^57^. Insufficient BP control under high-dose antihypertensive fixed-dose combination, potential contraindications, side effects or the presence of clinical alarm signs or symptoms, triggered the CDSS application to recommend referral to the health facility for professional care. Participants were free to accept or refuse the services offered by the lay CHW at any time. Participants not eligible for lay CHW-led care or refusing it were referred to their closest health facility for further management with bimonthly follow-ups by the lay CHW at the community level.

All data were collected by lay CHWs during home visits. During follow-up visits, lay CHWs inquired about the occurrence of possible adverse events. In addition, lay CHWs solicited adverse events through reporting by participants, friends or relatives, screening of participants’ personal health booklet and reporting by routine health facility staff any time during the follow-up period.

Adverse events reported by the lay CHWs were followed-up by supervising study staff to collect further clinical information. The pseudonymized reports were submitted to the study physician who classified the reports as SAE, adverse events of special interest (AESI) or neither of the two.

### Outcomes

The primary endpoint was BP control rate (proportion of participants with BP <140/90 mm Hg) 12 months after enrollment. Secondary endpoints were the BP control rate after 6 months; mean SBP; mean DBP; engagement in care (proportion of participants reporting intake of antihypertensive medication or reaching treatment targets without intake of medication); linkage to care (proportion of participants not taking treatment at enrollment who have initiated antihypertensive treatment during follow-up); self-reported adherence to antihypertensive treatment; 10-year cardiovascular risk using the WHO cardiovascular disease risk prediction tool^59^; BMI; abdominal circumference; blood lipid status; physical activity using the International Physical Activity Questionnaire Short Form (IPAQ-SF)^60^; alcohol consumption, tobacco use and dietary habits using a shortened unquantified food frequency questionnaire adapted from an assessment tool for obesity used in South Africa^61^; and fruit, vegetable and salt consumption using the WHO steps instrument^62^ 6 and 12 months after enrollment (see footnotes of Extended Data Table 1 for details). Adherence to antihypertensive treatment was assessed by asking how many of the last 4 days a dose of antihypertensive medication was missed. Participants reporting no doses missed over the last 4 days were considered adherent. Safety outcomes were the occurrence of SAEs and AESIs within 6 and 12 months after enrollment. AESIs were defined as adverse events consistent with hypertension complications, such as myocardial infarction, stroke, heart failure or chronic kidney disease, as well as adverse events related to the intake of antihypertensive medication leading to the discontinuation of the medication concerned. Predefined exploratory endpoints reported are the proportion of participants with elevated cardiovascular risk who are taking statin treatment and the proportion of participants in the intervention arm not accessing CHW-led hypertension care, and for what reason. Further predefined exploratory endpoints, that will be reported separately, were the number of consultations at a health facility and with a CHW, the proportion of participants who stop or interrupt antihypertensive treatment, the proportion of participants who reach treatment targets (<140/90 mm Hg) without intake of antihypertensive medication, the quality indicators of CHW activities (completeness of data and adherence to clinical algorithms), the causes for stopping or interruption of antihypertensive treatment, the health system and individual costs, cardiovascular risk estimated using different risk calculators, quality of life, access to care and medication, the type and dosage of antihypertensive medications and the number of participants with grade III hypertension. In addition to the prespecified endpoints, the change in mean SBP and DBP compared to baseline is reported to allow for comparison with other relevant trials in the field.

For all endpoints measured after 6 months, a window of 150 to 240 days after enrollment was applied, and for 12 month endpoints, a window of 300 to 420 days after enrollment was applied.

### Sample size calculations

The sample size was calculated assuming an individual randomization inflated by a design effect that accounts for variation at the cluster level, according to Rotondi and Donner^63^. On the basis of preliminary results from a noncommunicable disease prevalence survey in Lesotho^41^, we expected an adult prevalence of hypertension of 18%, with 55% having BP levels ≥140/90 mm Hg (ref. ^7^). Considering an average village size of 100 adult inhabitants, the average cluster size of eligible participants was estimated at 8. Assuming that 75% would accept the CHW care intervention, a probability of BP control of 60% among participants accepting the intervention and of 30% among those refusing it, we estimated an overall BP control rate of 52.5% in the intervention arm. We further assumed an intracluster correlation of 0.015 (refs. ^64,65^), a mean cluster size of 8 (s.d. of 5) and a probability of BP control of 35% in the control group^8^. A minimal sample size of 304 (152 in each arm and 19 clusters per study arm) was required to detect superiority with a type I error of 0.025 and a statistical power of 80%. Given that the ComBaCaL cohort includes 103 villages with an expected 8 persons with uncontrolled hypertension per village, we anticipated to exceed the minimum sample size of 304 participants. Owing to operational reasons and the uncertainty of some of the estimates, we decided to recruit in all 103 ComBaCaL cohort villages, for an anticipated sample size of 824 participants.

### Statistical analysis

Analyses were performed following the principles for analysis of cluster-randomized trials in health research as outlined by Donner and Klar^66^. The primary hypothesis was assessed in a modified ITT analysis set, including all participants as randomized except those with pregnancy during the follow-up. For the primary outcome, we used a mixed-effect logistic regression model adjusted for stratification factors, sex, age, district and access to health facilities with a random intercept at the cluster level.

Full adherence to the offered care package was not expected and therefore no strict per-protocol analysis was performed. However, to approximate a per-protocol estimate, we prespecified a safety set considering only participants with at least one hypertension care visit by the lay CHW in the intervention arm and at least one hypertension care visit at the health facility in the control arm. We conducted a predefined complete case analysis only including participants with available endpoint measurements. We estimated marginal predicted probabilities for SBP and DBP over time on the basis of the secondary analysis model (linear mixed-effects model with BP as outcome, cluster as random effect, covariate-adjusted as the primary model, but including baseline BP), and derived 95% CIs using 1,000 bootstrap samples. In addition, changes in SBP and DBP compared to baseline were modeled using the same adjusted mixed-effects model including 2.5th and 97.5th percentiles of predictions on 1,000 bootstrap samples.

For analyses of secondary outcomes, we used mixed-effects logistic or linear regression models adjusted for baseline outcomes in addition to stratification factors, without formal testing. Whenever a variance component of the mixed effects was estimated as negligeable and led to singularity diagnostics, we simplified the model by omitting the corresponding random effect rather than excluding clusters from the analysis. SAEs, AESIs, adherence to antihypertensive treatment and exploratory outcomes were analyzed descriptively. Modification of the intervention effect on the primary endpoint was assessed for the following predefined factors: eligibility for pharmacological treatment by the lay CHW (excluding participants requiring three or more antihypertensive drugs or having an intolerance against amlodipine or hydrochlorothiazide), age, sex, engagement in antihypertensive care at baseline, engagement in HIV care at baseline and access to health facility. Only if the *P* values of an interaction term were found to be below 0.1, formal subgroup analyses and subgroup credibility assessments are conducted.

Data management was done using Stata IC version 16.0 and data analysis using R version 4.3.3 (refs. ^67,68^).

### Inclusion and ethics statement

The intervention was developed in collaboration with local community members and the Lesotho Ministry of Health on the basis of a local noncommunicable diseases prevalence survey and burden assessment^7,8^ and a scoping literature review on community-based hypertension care in southern Africa^32^. Community advisory boards consisting of patient representatives, CHWs, village authorities, local health facility staff and local Ministry of Health administrative staff offered advice on the implementation of the larger ComBaCaL project, including this study. Representatives of both community advisory boards are part of the ComBaCaL project steering committee and involved in strategic decision-making. The study addressed an issue of high local relevance. Given the remoteness of most villages, poor transport infrastructure and limited health system resources, community-based health service delivery has been a cornerstone of the Lesotho health system for decades. There is a growing number of people with hypertension and other chronic conditions and the inclusion of noncommunicable disease services are considered a priority area in the Lesotho Community-Based Health Services Strategy^69^. It is a guiding principle of the project to align research activities with the local needs and to ensure local impact beyond the generation of novel scientific evidence through capacity building, collaboration, community involvement and health system strengthening activities.

Before the start of the study, the intervention was piloted in ten villages outside the ComBaCaL cohort in both study districts. Feedback from community members and CHWs guided the design and implementation of the final trial intervention^70,71^. Local researchers have been involved throughout the research process and share authorship. The local and regional research relevant to the study have been included in citations.

### Ethics and approval

The trial was approved by the National Health Research and Ethics Committee of Lesotho (ID 102-2022). In addition, the Ethikkomission Nordwest- und Zentralschweiz in Switzerland provided a statement confirming the trial meets all ethical requirements for a Swiss research project (ID AO_2022_00074).

The trial was nested within the ComBaCaL cohort study following the TwiCs design^34,35,52^. Following the French consent pattern of the TwiCs design^52^, the consent for the ComBaCaL cohort included consent to being randomized as part of future nested trials. The consent form is available as a supplementary file of the ComBaCaL cohort profile publication^34^ and on clinicaltrials.gov (NCT05596773).

Consent was obtained at three levels. First, oral consent from the village chiefs was obtained during village eligibility assessment prior to the CHW selection and enrollment of a village into the ComBaCaL cohort. Second, oral consent was obtained from the household head prior to the registration of a household in the ComBaCaL cohort data base and to approaching household members for individual consent. Third, individual written consent was obtained using a tablet-based consent application^35^. On the basis of participants’ characteristics (age, literacy and need for a guardian), the consent application displayed appropriate simplified study information and allowed for electronic signature on the informed consent form. Illiterate participants confirmed informed consent by drawing a cross in the electronic signature field, countersigned by an impartial witness. Signed consent forms were uploaded in the study database of the CDSS application and checked for correctness and completeness by the study team remotely and reconsent tasks were triggered via the CDSS application in case of errors.

Consent can be withdrawn any time on individual or household level. Pseudo-anonymized data collected until the time of withdrawal is retained in the database.

Following the TwiCs design, no trial-specific consent was required after identification of trial-eligible ComBaCaL cohort participants, and eligible cohort participants were automatically enrolled into the trial after eligibility screening. Participants in intervention villages were offered the intervention services by their lay CHW, including the offer of antihypertensive medication, and could then accept or decline any of the offered services (oral intervention consent).

Details on the design and consent procedures of the ComBaCaL cohort and nested trials have been published previously^34–36^.

### Reporting

Further information on research design is available in the Nature Portfolio Reporting Summary in the Supplementary Information. This study was conducted and reported according to the Consolidated Standards of Reporting Trials guidelines 2010 extension to cluster randomized trials^72^. The checklist is available in the Supplementary Information.

### Reporting summary

Further information on research design is available in the Nature Portfolio Reporting Summary linked to this article.

## Online content

Any methods, additional references, Nature Portfolio reporting summaries, source data, extended data, supplementary information, acknowledgements, peer review information; details of author contributions and competing interests; and statements of data and code availability are available at https://doi.org/10.1038/s41591-026-04208-w.

## Supplementary information

Supplementary Information Supplementary Tables 1–3 and Figs. 1 and 2.

Reporting Summary

Peer Review File

## Figures and Tables

**Extended Data Table 1:**
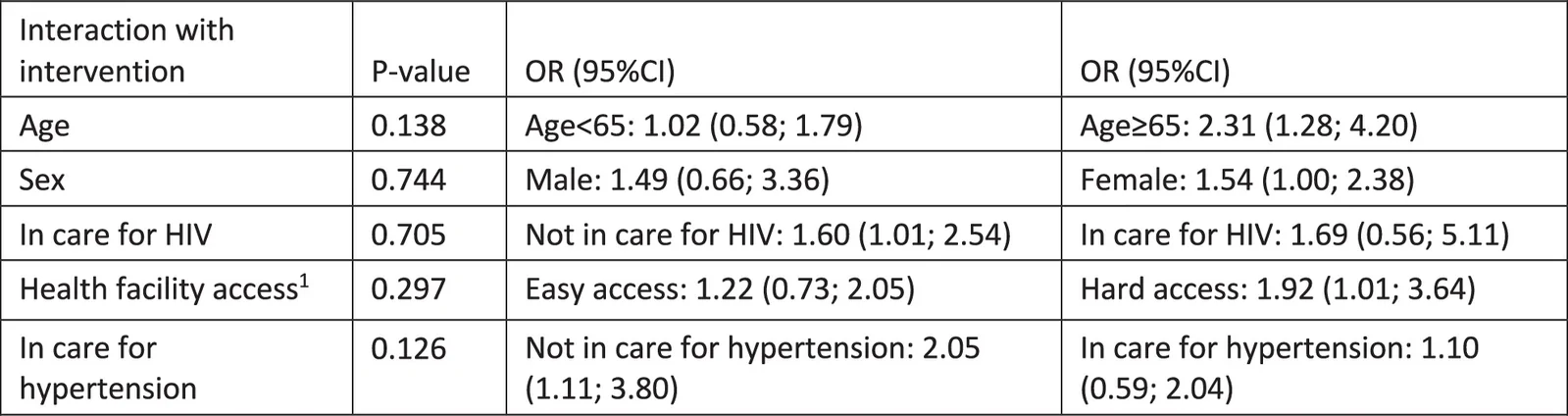
Interaction with the intervention of prespecified potential effect modifiers. OR: odds ratio

OR: odds ratio

1: Hard access defined as needing to cross a mountain or river or travel >10 km to the nearest health facility

**Extended Data Table 2:**
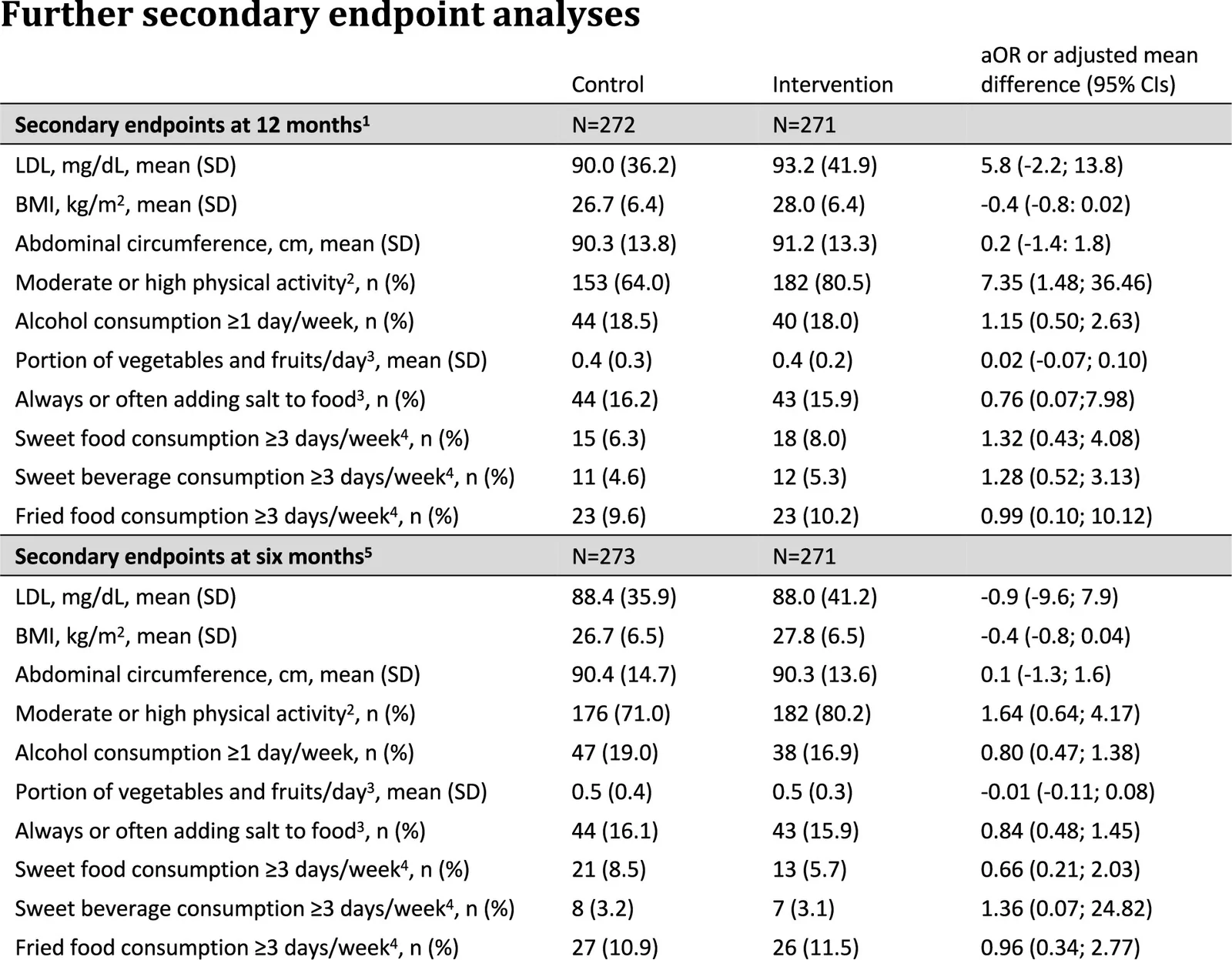
Intervention effects on further secondary endpoints

1: Four participants excluded due to pregnancy between enrollment and 12 months

2: Self-reported physical activity using the International Physical Activity Questionnaire Short Form (IPAQ-S)

3: Assessed using the WHO STEPS instrument

4: Assessed using a shortened unquantified food frequency questionnaire adapted from an assessment tool for obesity used in South Africa. For reporting of consumption of sweet food items, sweet beverage items and fried food items in the main manuscript tables, the questions were grouped (sweet food items: candy, biscuits/muffins, chocolate; sweet beverage items: fruit juices, carbonated soft drinks; fried food items: crisp, fries, makoenya). For each category the highest frequency of consumption of any of the items included in the category was reported.

5: Three participants excluded due to pregnancy between enrollment and 6 months

Abbreviations: aOR: adjusted odds ratio; CI: confidence interval; SD: standard deviation; BMI: body-mass index; LDL: low-density lipoprotein

Missing data at twelve months: LDL: 58, 7; BMI: 52, 48; abdominal circumference: 48, 56; physical activity: 33, 45; alcohol consumption: 34, 49; vegetables and fruit consumption: 33, 45; sweet food consumption: 33, 45; sweet beverage consumption: 33, 45; fried food consumption: 33, 45.

Missing data at six months: LDL: 62, 73; BMI: 34, 43; abdominal circumference: 32, 40; physical activity: 25, 44; alcohol consumption: 25, 46; vegetables and fruit consumption: 25, 44; sweet food consumption: 25, 44; sweet beverage consumption: 25, 44; fried food consumption: 25, 44.

## Data Availability

A de-identified individual-level key dataset alongside the statistical code for reproducing the primary and secondary endpoints is openly available from publication until at least 25 years thereafter via Zenodo at https://doi.org/10.5281/zenodo.16903262 (ref. ^73^). The trial was registered with ClinicalTrials.gov (NCT05684055), where a full protocol and statistical analysis plan are available. A study protocol manuscript has been published previously^36^. Requests for access to more detailed data may be made to the corresponding author by submitting a proposal. The proposal will be reviewed by the corresponding author and access granted if deemed reasonable within 4 weeks.

## Code availability

The code for the CDSS application used by CHWs for data collection and service delivery is available via GitHub at https://github.com/clinepi-usb/cht-combacal. Access to the test environment of the CDSS application is available from the corresponding author upon written request. The request will be reviewed by the corresponding author and credentials for the test environment shared if deemed reasonable within 4 weeks.
