# Supplementary material for: Lay community health worker-led care with mobile decision support for uncontrolled hypertension: a cluster-randomized trial
Source: Nat Med. 2026 Feb 12;32(3):915–23. doi: 10.1038/s41591-026-04208-w (PMC13768179; doi:10.1038/s41591-026-04208-w)
Supplement: Supplementary file 1 [file 41591_2026_4208_MOESM1_ESM.pdf]

nature medicine

Supplementary information https://doi.org/10.1038/s41591-026-04208-w

Lay community health worker-led care with mobile decision support for uncontrolled hypertension: a cluster-randomized trial

In the format provided by the authors and unedited

Supplementary information

Additional baseline characteristics

Participants, n	274	273	547
Cardiovascular disease risk[1], n (%)			
<5%	78 (30.0%)	50 (20.4%)	128 (25.3%)
5-10%	51 (19.6%)	45 (18.4%)	96 (19.0%)
10-20%	70 (26.9%)	87 (35.5%)	157 (31.1%)
20-30%	34 (13.1%)	39 (15.9%)	73 (14.5%)
≥30%	27 (10.4%)	24 (9.8%)	51 (10.1%)
Missing	14	28	42
Alcohol consumption			
Never	189 (69.2%)	193 (71.0%)	382 (70.1%)
Occasionally	21 (7.7%)	17 (6.2%)	38 (7.0%)
1 to 2 days per week	33 (12.1%)	31 (11.4%)	64 (11.7%)
≥3 days per week	30 (11.0%)	31 (11.4%)	61 (11.2%)
Missing	1	1	2
LDL cholesterol (mg/dl), mean (SD)	84.6 (36.8)	85.1 (37.2)	84.8 (37.0)
HDL (mg/dL), mean (SD)	40.0 (14.7)	43.6 (16.9)	41.7 (15.9)
Triglycerides, mean (SD)	156.4 (89.1)	153.1 (86.5)	154.8 (87.8)
Total cholesterol/HDL ratio, mean (SD)	4.1 (1.6)	3.9 (1.5)	4.0 (1.5)
MET minutes/week[2], mean (SD)	5099 (3857)	6384 (4513)	5737 (4241)
Adding salt while eating[3], n (%)			
Always	37 (13.5%)	37 (13.6%)	74 (13.6%)
Often	8 (2.9%)	7 (2.6%)	15 (2.7%)
Sometimes	72 (26.3%)	43 (15.8%)	115 (21.1%)
Never	157 (57.3%)	185 (68.0%)	342 (62.6%)
Missing	0	1	1
Adding salt during cooking[3], n (%)	154 (56.2%)	201 (73.6%)	355 (64.9%)
Always	6 (2.2%)	1 (0.4%)	7 (1.3%)
Often	55 (20.1%)	26 (9.5%)	81 (14.8%)
Sometimes	59 (21.5%)	45 (16.5%)	104 (19.0%)
Never	0	0	0
Missing			
Eating processed food high in salt[3], n (%)			
Always	23 (8.4%)	16 (5.9%)	39 (7.1%)
Often	8 (2.9%)	11 (4.0%)	19 (3.5%)
Sometimes	86 (31.4%)	61 (22.3%)	147 (26.9%)
Never	157 (57.3%)	185 (67.8%)	342 (62.5%)
Missing	0	0	0
Perceived quantity of salt consumption[3], n (%)			
Far too much	3 (1.1%)	1 (0.4%)	4 (0.7%)
Too much	9 (3.3%)	10 (3.7%)	19 (3.5%)
Right amount	157 (57.7%)	153 (57.1%)	310 (57.4%)
Too little	94 (34.6%)	92 (34.3%)	186 (34.4%)

Far too little	9 (3.3%)	12 (4.5%)	21 (3.9%)
Missing	2	5	7
Perceived importance of lowering salt[3], n (%)			
Very important	247 (94.6%)	249 (94.3%)	496 (94.5%)
Somewhat important	6 (2.3%)	10 (3.8%)	16 (3.0%)
Not important	8 (3.1%)	5 (1.9%)	13 (2.5%)
Missing	13	9	22
Believing that too much salt causes health problems[3], n (%)			
Yes	241 (92.0%)	235 (89.4%)	476 (90.7%)
No	21 (8.0%)	28 (10.6%)	49 (9.3%)
Missing	12	10	22
Fruits/day[3], mean (SD)	0.3 (0.5)	0.3 (0.5)	0.3 (0.5)
Vegetables/day[3], mean (SD)	0.3 (0.2)	0.3 (0.2)	0.3 (0.2)
Portion of vegetables and fruits/day, mean (SD)	0.6 (0.6)	0.6 (0.5)	0.6 (0.5)
Sweet food consumption ≥3 days/week[4], n (%)	20 (7.3)	37 (13.6)	57 (10.4)
Sweet beverage consumption ≥3 days/week[4], n (%)	20 (7.3)	19 (7.0)	39 (7.1)
Fried food consumption ≥3 days/week[4], n (%)	40 (14.6)	60 (22.0)	100 (18.3)
Days fruit juice consumed/week[4], n (%)			
Less than one day	171 (62.6%)	192 (70.8%)	363 (66.7%)
1-2 days	87 (31.9%)	64 (23.6%)	151 (27.8%)
3-4 days	8 (2.9%)	10 (3.7%)	18 (3.3%)
5 or more days	7 (2.6%)	5 (1.8%)	12 (2.2%)
Missing	1	2	3
Days carbonated soft drink consumed/week[4], n (%)			
Less than one day	203 (74.4%)	193 (71.2%)	396 (72.8%)
1-2 days	61 (22.3%)	70 (25.8%)	131 (24.1%)
3-4 days	7 (2.6%)	6 (2.2%)	13 (2.4%)
5 or more days	2 (0.7%)	2 (0.7%)	4 (0.7%)
Missing	1	2	3
Days candy consumed/week[4], n (%)			
Less than one day	171 (62.4%)	156 (57.6%)	327 (60.0%)
1-2 days	89 (32.5%)	80 (29.5%)	169 (31.0%)
3-4 days	9 (3.3%)	19 (7.0%)	28 (5.1%)
5 or more days	5 (1.8%)	16 (5.9%)	21 (3.9%)
Missing	0	2	2
Days biscuits or muffins consumed/week[4], n (%)			
Less than one day	222 (81.0%)	211 (77.9%)	433 (79.4%)
1-2 days	44 (16.1%)	56 (20.7%)	100 (18.3%)
3-4 days	4 (1.5%)	3 (1.1%)	7 (1.3%)
5 or more days	4 (1.5%)	1 (0.4%)	5 (0.9%)
Missing	0	2	2
Days chocolate consumed/week[4], n (%)			
Less than one day	233 (85.0%)	231 (85.2%)	464 (85.1%)
1-2 days	34 (12.4%)	38 (14.0%)	72 (13.2%)
3-4 days	6 (2.2%)	1 (0.4%)	7 (1.3%)
5 or more days	1 (0.4%)	1 (0.4%)	2 (0.4%)

Missing	0	2	2
Days crisps consumed/week[4], n (%)			
Less than one day	151 (55.1%)	147 (54.2%)	298 (54.7%)
1-2 days	102 (37.2%)	95 (35.1%)	197 (36.1%)
3-4 days	18 (6.6%)	20 (7.4%)	38 (7.0%)
5 or more days	3 (1.1%)	9 (3.3%)	12 (2.2%)
Missing	0	2	2
Days fries consumed/week[4], n (%)			
Less than one day	183 (66.8%)	177 (65.3%)	360 (66.1%)
1-2 days	75 (27.4%)	75 (27.7%)	150 (27.5%)
3-4 days	13 (4.7%)	18 (6.6%)	31 (5.7%)
5 or more days	3 (1.1%)	1 (0.4%)	4 (0.7%)
Missing	0	2	2
Days makoenya[5] consumed/week[4], n (%)			
Less than one day	144 (52.6%)	136 (50.0%)	280 (51.3%)
1-2 days	110 (40.1%)	105 (38.6%)	215 (39.4%)
3-4 days	17 (6.2%)	25 (9.2%)	42 (7.7%)
5 or more days	3 (1.1%)	6 (2.2%)	9 (1.6%)
Missing	0	1	1

Supplementary Table 1 Additional baseline characteristics.

Supplementary Table 1 legend

1: 10-year risk for a cardiovascular event, using the WHO laboratory-based cardiovascular risk calculator
2: Self-reported physical activity using the International Physical Activity Questionnaire Short Form (IPAQ-S)
3: Assessed using the WHO STEPS instrument
4: Assessed using a shortened unquantified food frequency questionnaire adapted from an assessment tool for obesity used in South Africa. For reporting of consumption of sweet food items, sweet beverage items and fried food items in the main manuscript tables, the questions were grouped (sweet food items: candy, biscuits/muffins, chocolate; sweet beverage items: fruit juices, carbonated soft drinks; fried food items: crisp, fries, makoenya). For each category the highest frequency of consumption of any of the items included in the category was reported.
5: Traditional fried bread bun

Abbreviations: SD: standard deviation, LDL: low-density lipoprotein, HDL: high-density lipoprotein, MET: metabolic equivalent of task
Missing data: Cardiovascular disease risk: 14 control, 28 intervention; LDL cholesterol: 25, 57; HDL: 14, 28; triglycerides: 14, 28; Total cholesterol/HDL ratio: 33, 54; MET minutes/week: 0, 3;

Additional outcome data after twelve months

Participants[1], n	272	271	543
Cardiovascular disease risk[2], n (%)			
<5%	67 (28.3%)	56 (24.9%)	123 (26.6%)
5-10%	52 (21.9%)	44 (19.6%)	96 (20.8%)
10-20%	66 (27.8%)	76 (33.8%)	142 (30.7%)
20-30%	29 (12.2%)	32 (14.2%)	61 (13.2%)
≥30%	23 (9.7%)	17 (7.6%)	40 (8.7%)
Missing	35	46	81
Alcohol consumption			
Never	166 (69.7%)	155 (69.8%)	321 (69.8%)
Occasionally	28 (11.8%)	27 (12.2%)	55 (12.0%)
1 to 2 days per week	21 (8.8%)	18 (8.1%)	39 (8.5%)
≥3 days per week	23 (9.7%)	22 (9.9%)	45 (9.8%)
Missing	34	49	83
HDL (mg/dL), mean (SD)	44.0 (17.8)	48.1 (20.6)	46.0 (19.3)
Triglycerides, mean (SD)	165.7 (98.2)	141.2 (98.7)	153.8 (99.1)
Total cholesterol/HDL ratio, mean (SD)	4.1 (1.4)	3.7 (1.5)	3.9 (1.5)
MET minutes/week[3], mean (SD)	3614 (3394)	5197 (3946)	4384 (3753)
Low physical activity[3]	86 (36.0)	44 (19.5)	130 (28.0)
Moderate or high physical activity[3]	153 (64.0)	182 (80.5)	335 (72.0)
Missing	33	45	78
Adding salt while eating[4], n (%)			
Always	29 (12.1%)	19 (8.4%)	48 (10.3%)
Often	6 (2.5%)	7 (3.1%)	13 (2.8%)
Sometimes	89 (37.2%)	65 (28.8%)	154 (33.1%)
Never	115 (48.1%)	135 (59.7%)	250 (53.8%)
Missing	33	45	78
Adding salt during cooking[4], n (%)			
Always	111 (46.4%)	102 (45.1%)	213 (45.8%)
Often	7 (2.9%)	4 (1.8%)	11 (2.4%)
Sometimes	63 (26.4%)	57 (25.2%)	120 (25.8%)
Never	58 (24.3%)	63 (27.9%)	121 (26.0%)
Missing	33	45	78
Eating processed food high in salt[4], n (%)			
Always	12 (5.0%)	18 (8.0%)	30 (6.5%)
Often	6 (2.5%)	8 (3.6%)	14 (3.0%)
Sometimes	111 (46.4%)	72 (32.0%)	183 (39.4%)
Never	110 (46.0%)	127 (56.4%)	237 (51.1%)
Missing	33	46	79
Perceived quantity of salt consumption[4], n (%)			
Far too much	1 (0.4%)	5 (2.3%)	6 (1.3%)
Too much	6 (2.5%)	8 (3.7%)	14 (3.1%)
Right amount	164 (69.5%)	122 (55.7%)	286 (62.9%)
Too little	57 (24.2%)	79 (36.1%)	136 (29.9%)

Far too little	8 (3.4%)	5 (2.3%)	13 (2.9%)
Missing	36	52	88
Perceived importance of lowering salt[4], n (%)			
Very important	227 (96.6%)	220 (97.8%)	447 (97.2%)
Somewhat important	8 (3.4%)	3 (1.3%)	11 (2.4%)
Not important	0 (0.0%)	2 (0.9%)	2 (0.4%)
Missing	37	46	83
Believing that too much salt causes health problem[4], n (%)			
Yes	220 (93.2%)	201 (89.7%)	421 (91.5%)
No	16 (6.8%)	23 (10.3%)	39 (8.5%)
Missing	36	47	83
Fruits/day[4], mean (SD)	0.1 (0.2)	0.1 (0.1)	0.1 (0.1)
Vegetables/day[4], mean (SD)	0.3 (0.2)	0.3 (0.2)	0.3 (0.2)
Days fruit juice consumed/week[5], n (%)			
Less than one day	173 (72.4%)	166 (73.8%)	339 (73.1%)
1-2 days	57 (23.8%)	54 (24.0%)	111 (23.9%)
3-4 days	6 (2.5%)	5 (2.2%)	11 (2.4%)
5 or more days	3 (1.3%)	0 (0.0%)	3 (0.6%)
Missing	33	46	79
Days carbonated soft drink consumed/week[5], n (%)			
Less than one day	188 (78.7%)	178 (78.8%)	366 (78.7%)
1-2 days	46 (19.2%)	40 (17.7%)	86 (18.5%)
3-4 days	4 (1.7%)	8 (3.5%)	12 (2.6%)
5 or more days	1 (0.4%)	0 (0.0%)	1 (0.2%)
Missing	33	45	78
Days candy consumed/week[5], n (%)			
Less than one day	163 (68.2%)	147 (65.0%)	310 (66.7%)
1-2 days	66 (27.6%)	66 (29.2%)	132 (28.4%)
3-4 days	10 (4.2%)	10 (4.4%)	20 (4.3%)
5 or more days	0 (0.0%)	3 (1.3%)	3 (0.6%)
Missing	33	45	78
Days biscuits or muffins consumed/week[5], n (%)			
Less than one day	199 (83.3%)	178 (78.8%)	377 (81.1%)
1-2 days	34 (14.2%)	42 (18.6%)	76 (16.3%)
3-4 days	5 (2.1%)	5 (2.2%)	10 (2.2%)
5 or more days	1 (0.4%)	1 (0.4%)	2 (0.4%)
Missing	33	45	78
Days chocolate consumed/week[5], n (%)			
Less than one day	216 (90.4%)	205 (90.7%)	421 (90.5%)
1-2 days	17 (7.1%)	18 (8.0%)	35 (7.5%)
3-4 days	5 (2.1%)	2 (0.9%)	7 (1.5%)
5 or more days	1 (0.4%)	1 (0.4%)	2 (0.4%)
Missing	33	45	78
Days crisps consumed/week[5], n (%)			
Less than one day	154 (64.7%)	132 (58.4%)	286 (61.6%)
1-2 days	74 (31.1%)	80 (35.4%)	154 (33.2%)
3-4 days	8 (3.4%)	12 (5.3%)	20 (4.3%)

5 or more days	2 (0.8%)	2 (0.9%)	4 (0.9%)
Missing	34	45	79
Days fries consumed/week[5], n (%)			
Less than one day	191 (79.9%)	181 (80.1%)	372 (80.0%)
1-2 days	38 (15.9%)	43 (19.0%)	81 (17.4%)
3-4 days	8 (3.3%)	2 (0.9%)	10 (2.2%)
5 or more days	2 (0.8%)	0 (0.0%)	2 (0.4%)
Missing	33	45	78
Days makoenya[6] consumed/week[5], n (%)			
Less than one day	143 (59.8%)	143 (63.3%)	286 (61.5%)
1-2 days	81 (33.9%)	72 (31.9%)	153 (32.9%)
3-4 days	10 (4.2%)	9 (4.0%)	19 (4.1%)
5 or more days	5 (2.1%)	2 (0.9%)	7 (1.5%)
Missing	33	45	78

Supplementary Table 2 Additional outcomes after twelve months.

Supplementary Table 2 legend

1: Four participants excluded due to pregnancy between enrolment and 12 months
2: 10-year risk for a cardiovascular event, using the WHO laboratory-based cardiovascular risk calculator.
3: Self-reported physical activity using the International Physical Activity Questionnaire Short Form (IPAQ-S).
4: Assessed using the WHO steps instrument.
5: Assessed using a shortened unquantified food frequency questionnaire adapted from an assessment tool for obesity used in South Africa. For reporting of consumption of sweet food items, sweet beverage items and fried food items in the main manuscript tables, the questions were grouped (sweet food items: candy, biscuits/muffins, chocolate; sweet beverage items: fruit juices, carbonated soft drinks; fried food items: crisp, fries, makoenya). For each category the highest frequency of consumption of any of the items included in the category was reported.
6: Traditional fried bread bun

Additional outcome data after six months

Participants[1], n	273	271	544
Cardiovascular disease risk[2], n (%)			
<5%	68 (28.5%)	51 (23.4%)	119 (26.0%)
5-10%	54 (22.6%)	52 (23.9%)	106 (23.2%)
10-20%	69 (28.9%)	72 (33.0%)	141 (30.9%)
20-30%	29 (12.1%)	28 (12.8%)	57 (12.5%)
≥30%	19 (7.9%)	15 (6.9%)	34 (7.4%)
Missing	34	53	87
Alcohol consumption			
Never	170 (68.5%)	160 (71.1%)	330 (69.8%)
Occasionally	31 (12.5%)	27 (12.0%)	58 (12.3%)
1 to 2 days per week	26 (10.5%)	18 (8.0%)	44 (9.3%)
≥3 days per week	21 (8.5%)	20 (8.9%)	41 (8.7%)
Missing	25	46	71
HDL (mg/dL), mean (SD)	38.7 (16.3)	45.9 (19.2)	42.1 (18.1)
Triglycerides, mean (SD)	146.8 (80.5)	147.4 (90.6)	147.1 (85.4)
Total cholesterol/HDL ratio, mean (SD)	4.3 (1.6)	3.9 (1.5)	4.1 (1.6)
MET minutes/week[3], mean (SD)	3996 (3516)	5802 (4411)	4859 (4066)
Low physical activity[3]	72 (29.0)	45 (19.8)	117 (24.6)
Moderate or high physical activity[3]	176 (71.0)	182 (80.2)	358 (75.4)
Missing	25	44	69
Adding salt while eating[4], n (%)			
Always	31 (12.5%)	20 (8.8%)	51 (10.7%)
Often	5 (2.0%)	8 (3.5%)	13 (2.7%)
Sometimes	81 (32.7%)	54 (23.8%)	135 (28.4%)
Never	131 (52.8%)	145 (63.9%)	276 (58.1%)
Missing	25	44	69
Adding salt during cooking[4], n (%)			
Always	115 (46.4%)	102 (44.9%)	217 (45.7%)
Often	7 (2.8%)	11 (4.8%)	18 (3.8%)
Sometimes	78 (31.5%)	52 (22.9%)	130 (27.4%)
Never	48 (19.4%)	62 (27.3%)	110 (23.2%)
Missing	25	44	69
Eating processed food high in salt[4], n (%)			
Always	13 (5.3%)	20 (8.8%)	33 (7.0%)
Often	6 (2.4%)	7 (3.1%)	13 (2.7%)
Sometimes	95 (38.5%)	74 (32.6%)	169 (35.7%)
Never	133 (53.8%)	126 (55.5%)	259 (54.6%)
Missing	26	44	70
Perceived quantity of salt consumption[4], n (%)			
Far too much	3 (1.2%)	9 (4.0%)	12 (2.5%)
Too much	2 (0.8%)	9 (4.0%)	11 (2.3%)
Right amount	154 (62.9%)	128 (56.6%)	282 (59.9%)
Too little	81 (33.1%)	79 (35.0%)	160 (34.0%)
Far too little	5 (2.0%)	1 (0.4%)	6 (1.3%)

Missing	28	45	73
Perceived importance of lowering salt[4], n (%)			
Very important	240 (98.0%)	219 (97.3%)	459 (97.7%)
Somewhat important	4 (1.6%)	5 (2.2%)	9 (1.9%)
Not important	1 (0.4%)	1 (0.4%)	2 (0.4%)
Missing	28	46	74
Believing that too much salt causes health problem[4], n (%)			
Yes	219 (89.8%)	198 (88.8%)	417 (89.3%)
No	25 (10.2%)	25 (11.2%)	50 (10.7%)
Missing	29	48	77
Fruits/day[4], mean (SD)	0.224 (0.294)	0.195 (0.274)	0.210 (0.285)
Vegetables/day[4], mean (SD)	0.307 (0.200)	0.303 (0.178)	0.305 (0.190)
Days fruit juice consumed/week[5], n (%)			
Less than one day	172 (69.4%)	178 (78.8%)	350 (73.8%)
1-2 days	71 (28.6%)	44 (19.5%)	115 (24.3%)
3-4 days	4 (1.6%)	4 (1.8%)	8 (1.7%)
5 or more days	1 (0.4%)	0 (0.0%)	1 (0.2%)
Missing	25	45	70
Days carbonated soft drink consumed/week[5], n (%)			
Less than one day	192 (78.4%)	184 (81.1%)	376 (79.7%)
1-2 days	49 (20.0%)	40 (17.6%)	89 (18.9%)
3-4 days	2 (0.8%)	3 (1.3%)	5 (1.1%)
5 or more days	2 (0.8%)	0 (0.0%)	2 (0.4%)
Missing	28	44	72
Days candy consumed/week[5], n (%)			
Less than one day	167 (67.3%)	154 (68.1%)	321 (67.7%)
1-2 days	62 (25.0%)	61 (27.0%)	123 (25.9%)
3-4 days	15 (6.0%)	10 (4.4%)	25 (5.3%)
5 or more days	4 (1.6%)	1 (0.4%)	5 (1.1%)
Missing	25	45	70
Days biscuits or muffins consumed/week[5], n (%)			
Less than one day	217 (87.9%)	180 (79.3%)	397 (83.8%)
1-2 days	23 (9.3%)	43 (18.9%)	66 (13.9%)
3-4 days	5 (2.0%)	2 (0.9%)	7 (1.5%)
5 or more days	2 (0.8%)	2 (0.9%)	4 (0.8%)
Missing	26	44	70
Days chocolate consumed/week[5], n (%)			
Less than one day	220 (89.1%)	198 (87.6%)	418 (88.4%)
1-2 days	21 (8.5%)	26 (11.5%)	47 (9.9%)
3-4 days	5 (2.0%)	1 (0.4%)	6 (1.3%)
5 or more days	1 (0.4%)	1 (0.4%)	2 (0.4%)
Missing	26	45	71
Days crisps consumed/week[5], n (%)			
Less than one day	154 (62.1%)	134 (59.0%)	288 (60.6%)
1-2 days	82 (33.1%)	79 (34.8%)	161 (33.9%)
3-4 days	9 (3.6%)	14 (6.2%)	23 (4.8%)
5 or more days	3 (1.2%)	0 (0.0%)	3 (0.6%)

Missing	25	44	69
Days fries consumed/week[5], n (%)			
Less than one day	176 (71.3%)	181 (79.7%)	357 (75.3%)
1-2 days	62 (25.1%)	37 (16.3%)	99 (20.9%)
3-4 days	7 (2.8%)	6 (2.6%)	13 (2.7%)
5 or more days	2 (0.8%)	3 (1.3%)	5 (1.1%)
Missing	26	44	70
Days makoenya[6] consumed/week[5], n (%)			
Less than one day	138 (55.6%)	139 (61.2%)	277 (58.3%)
1-2 days	91 (36.7%)	77 (33.9%)	168 (35.4%)
3-4 days	14 (5.6%)	10 (4.4%)	24 (5.1%)
5 or more days	5 (2.0%)	1 (0.4%)	6 (1.3%)
Missing	25	44	69

Supplementary Table 3 Additional outcomes after six months.

Supplementary Table 3 legend
1: Three participants excluded due to pregnancy between enrolment and 6 months
2: 10-year risk for a cardiovascular event, using the WHO laboratory-based cardiovascular risk calculator
3: Self-reported physical activity using the International Physical Activity Questionnaire Short Form (IPAQ-S)
4: Assessed using the WHO steps instrument
5: Assessed using a shortened unquantified food frequency questionnaire adapted from an assessment tool for obesity used in South Africa. For reporting of consumption of sweet food items, sweet beverage items and fried food items in the main manuscript tables, the questions were grouped (sweet food items: candy, biscuits/muffins, chocolate; sweet beverage items: fruit juices, carbonated soft drinks; fried food items: crisp, fries, makoenya). For each category the highest frequency of consumption of any of the items included in the category was reported.
6: Traditional fried bread bun

Abbreviations: SD: standard deviation, LDL: low-density lipoprotein, HDL: high-density lipoprotein, MET: metabolic equivalent of task
Missing data: Cardiovascular disease risk: 34 control, 53 intervention; LDL cholesterol: 62, 73; HDL: 32, 52; triglycerides: 32, 52; Total cholesterol/HDL ratio: 62, 68; MET minutes/week: 25, 44;

Screening and diagnosis algorithm

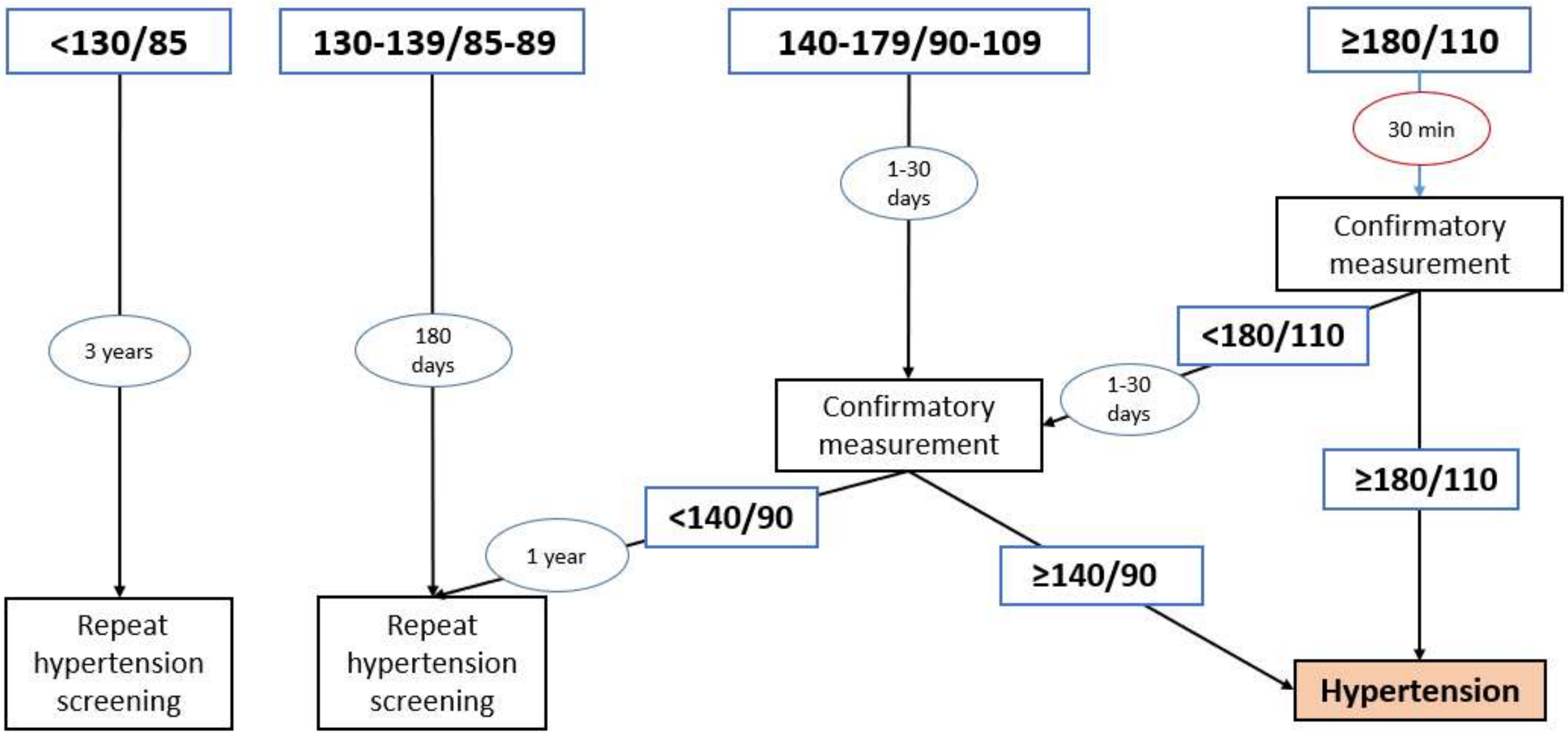

Supplementary information figure 1 Hypertension screening and diagnosis algorithm applied in all villages. People reporting the use of antihypertensive medication were considered as having hypertension independent of blood pressure values. People with hypertension and a blood pressure ≥140/90 were enrolled in the trial.

Intervention treatment algorithm

At every visit, the Community Health Worker (CHW) documented the current medication, adherence, and blood pressure (BP) values. If no contraindications were present, CHWs offered a fixed-dose combination pill containing amlodipine and hydrochlorothiazide with titration according to the algorithm of the Lesotho Standard Treatment Guidelines[1] (supplementary information fig. 2). Participants with a BP ≥140/90 mmHg under high-dose amlodipine/hydrochlorothiazide or with contraindications against amlodipine/hydrochlorothiazide or refusing medications offered by the CHW, were referred to the closest health facility. Follow-up visits for BP check-ups were scheduled after one months if the dose was adjusted during the index visit or after two months if the dose was not adjusted. Participants with high cardiovascular disease (CVD) risk, and no contraindications were offered atorvastatin 10mg daily. High CVD risk among people with hypertension was defined as having a history of stroke or myocardial infarction, having diabetes or chronic kidney disease or being ≥50 years old and currently smoking or having a BMI ≥ 30 kg/m^2.

Participants with a history of myocardial infarction or stroke were offered acetyl salicylic acid 100mg daily.

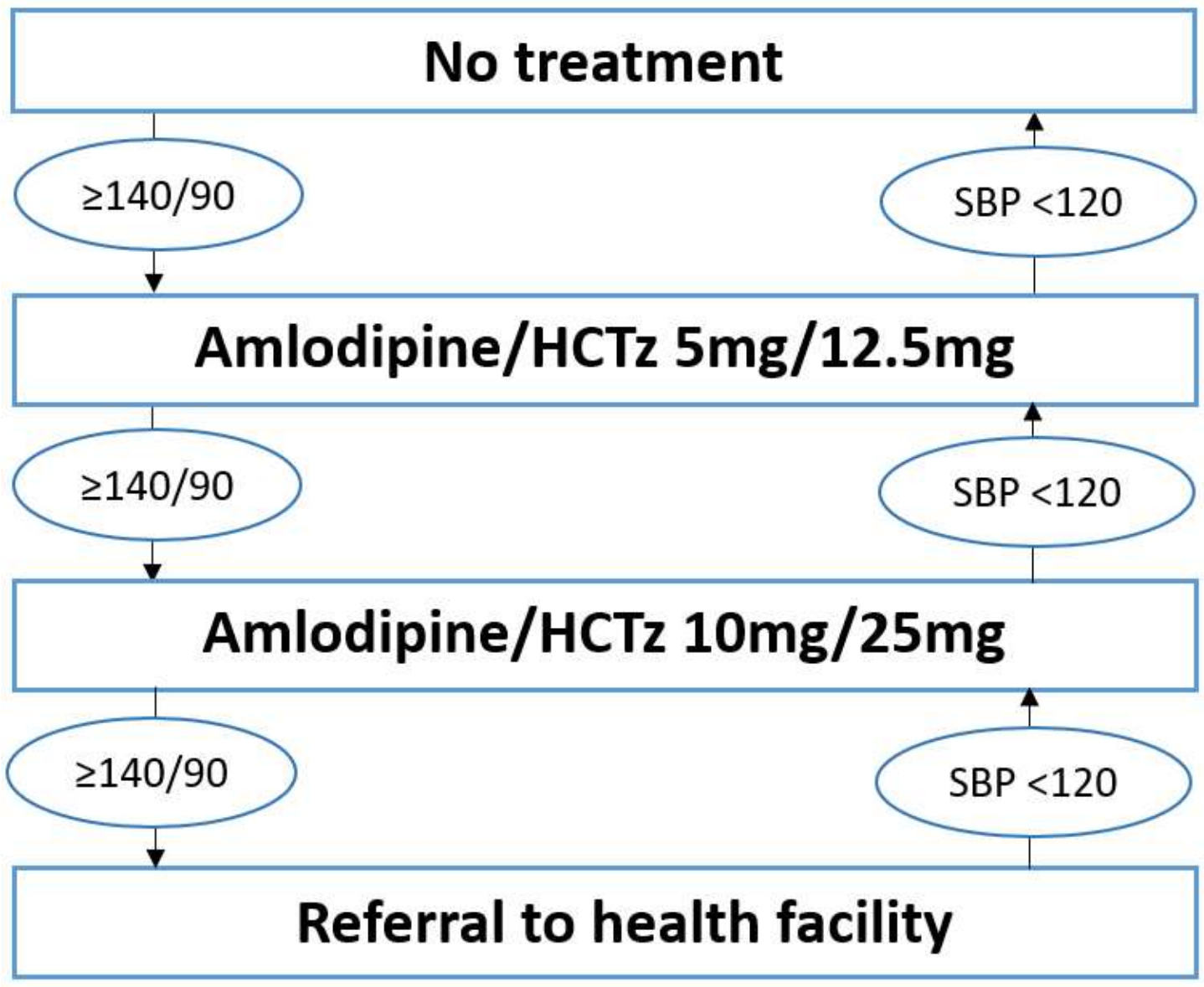

Supplementary information figure 2 Hypertension treatment algorithm applied by CHWs in the intervention villages. HCTz: hydrochlorothiazide; SBP: Systolic blood pressure.

Reference

1. Ministry of Health Lesotho. Standard Treatment Guidelines for Lesotho. (2022).
