# Supplementary material for: Lay community health worker-led care with mobile decision support for uncontrolled hypertension: a cluster-randomized trial
Source: Nat Med. 2026 Feb 12;32(3):915–23. doi: 10.1038/s41591-026-04208-w (PMC13768179; doi:10.1038/s41591-026-04208-w)
Supplement: Supplementary file 2 [file 41591_2026_4208_MOESM2_ESM.pdf]

nature portfolio

Corresponding author(s): Niklaus Labhardt, Felix Gerber
Last updated by author(s): Nov 2, 2025

Reporting Summary

Nature Portfolio wishes to improve the reproducibility of the work that we publish. This form provides structure for consistency and transparency in reporting. For further information on Nature Portfolio policies, see our Editorial Policies and the Editorial Policy Checklist.

Statistics

For all statistical analyses, confirm that the following items are present in the figure legend, table legend, main text, or Methods section.

n/a	Confirmed	
☐	☒	The exact sample size (*n*) for each experimental group/condition, given as a discrete number and unit of measurement
☐	☒	A statement on whether measurements were taken from distinct samples or whether the same sample was measured repeatedly
☐	☒	The statistical test(s) used AND whether they are one- or two-sided<br>*Only common tests should be described solely by name; describe more complex techniques in the Methods section.*
☐	☒	A description of all covariates tested
☐	☒	A description of any assumptions or corrections, such as tests of normality and adjustment for multiple comparisons
☐	☒	A full description of the statistical parameters including central tendency (e.g. means) or other basic estimates (e.g. regression coefficient) AND variation (e.g. standard deviation) or associated estimates of uncertainty (e.g. confidence intervals)
☐	☒	For null hypothesis testing, the test statistic (e.g. *F*, *t*, *r*) with confidence intervals, effect sizes, degrees of freedom and *P* value noted<br>*Give P values as exact values whenever suitable.*
☒	☐	For Bayesian analysis, information on the choice of priors and Markov chain Monte Carlo settings
☐	☒	For hierarchical and complex designs, identification of the appropriate level for tests and full reporting of outcomes
☒	☐	Estimates of effect sizes (e.g. Cohen's *d*, Pearson's *r*), indicating how they were calculated

Our web collection on statistics for biologists contains articles on many of the points above.

Software and code

Policy information about availability of computer code

Data collection	Data was collected by Community Health Workers through a data collection and clinical decision support application that was configured by the study team based on the open source Community Health Toolkit Core Framework (versions 3.12, 3.14, 4.6 and 4.13). The code for the application can be found at https://github.com/clinepi-usb/cht-combacal. Access to the test environment of the application is available from the corresponding author upon written request. The request will be reviewed by the corresponding author and credentials for the test environment shared if deemed reasonable within four weeks.
Data analysis	Data management was done using Stata IC 16.0 and data analysis using R version 4.3.3.

For manuscripts utilizing custom algorithms or software that are central to the research but not yet described in published literature, software must be made available to editors and reviewers. We strongly encourage code deposition in a community repository (e.g. GitHub). See the Nature Portfolio guidelines for submitting code & software for further information.

Data

Policy information about availability of data

All manuscripts must include a data availability statement. This statement should provide the following information, where applicable:

- Accession codes, unique identifiers, or web links for publicly available datasets
- A description of any restrictions on data availability
- For clinical datasets or third party data, please ensure that the statement adheres to our policy

A de-identified individual-level key dataset alongside the statistical code for reproducing the primary and secondary endpoints is openly available from publication until 25 years thereafter at https://doi.org/10.5281/zenodo.16903262. The trial was registered with ClinicalTrials.gov (NCT05684055), where a full protocol and statistical analysis plan are available. A study protocol manuscript has been published previously. Requests for access to more detailed data may be made to the corresponding author by submitting a proposal. The proposal will be reviewed by the corresponding author and access granted if deemed reasonable within four weeks.

Research involving human participants, their data, or biological material

Policy information about studies with human participants or human data. See also policy information about sex, gender (identity/presentation), and sexual orientation and race, ethnicity and racism.

Reporting on sex and gender	Only self-reported sex was collected. The proportion of male and female in the study population is reported. In a pre-specified analysis the interaction of sex with the intervention effect was assessed. As there was no relevant interaction of sex with the intervention effect, the subgroup analysis by sex is only provided in the extended data file but not in the main manuscript.
Reporting on race, ethnicity, or other socially relevant groupings	Race, ethnicity, or other socially relevant groupings were not collected in this study. The study population was recruitment among inhabitants of remote villages in rural Lesotho with no relevant ethnical or social subgroups.
Population characteristics	547 non-pregnant adults with uncontrolled hypertension living in a randomly selected study village in the rural areas of Butha-Buthe and Mokhotlong districts, Lesotho. Mean age was 62.3 years, mean systolic blood pressure 149.0 mmHg, mean diastolic blood pressure 95.0 mmHg, 75.0% female, 44.6% reported use of antihypertensive medication at baseline, 15.7% reported living with HIV.
Recruitment	Participants for this trial were recruited among participants of the overarching ComBaCaL cohort study that includes all consenting inhabitants of 103 randomly selected villages in rural Lesotho after home-based eligibility screening including blood pressure measurements.
Ethics oversight	The study was approved by the National Health Research and Ethics Committee (NH-REC) of Lesotho (ID 102-2022). Additionally, the Ethikkommission Nordwest- und Zentralschweiz (EKNZ) in Switzerland provided a statement confirming the trial meets all ethical requirements for a Swiss research project (ID AO_2022_00074).

Note that full information on the approval of the study protocol must also be provided in the manuscript.

Field-specific reporting

Please select the one below that is the best fit for your research. If you are not sure, read the appropriate sections before making your selection.

☒ Life sciences ☐ Behavioural & social sciences ☐ Ecological, evolutionary & environmental sciences

For a reference copy of the document with all sections, see nature.com/documents/nr-reporting-summary-flat.pdf

Life sciences study design

All studies must disclose on these points even when the disclosure is negative.

Sample size	The sample size was calculated assuming an individual randomization inflated by a design effect that accounts for variation at cluster level. Assumptions: hypertension prevalence: 18%, cluster size: 100 adults, BP control rate in control group: 35%; control rate in intervention group: 52.5%, intra-cluster correlation: 0.015, type 1 error of 0.025, power: 80%. 304 participants minimally required (38 clusters); decided to recruit across all 103 ComBaCaL cohort clusters due to operational reasons and uncertainties of some estimates.
Data exclusions	Participants who became pregnant during follow-up were excluded from the primary analysis.
Replication	Not applicable
Randomization	Half of the ComBaCaL cohort villages were randomly allocated 1:1 to intervention and control groups by a statistician not involved in the study. Randomization was stratified by district (Mokhotlong versus Butha-Buthe) and access to health facilities (easy versus difficult).

Blinding | Participants were blinded to the allocation, meaning that participants in the control villages were not aware of the intervention being implemented in other villages. CHWs who enrolled participants, provided the intervention and collected endpoint data were not blinded

Reporting for specific materials, systems and methods

We require information from authors about some types of materials, experimental systems and methods used in many studies. Here, indicate whether each material, system or method listed is relevant to your study. If you are not sure if a list item applies to your research, read the appropriate section before selecting a response.

Materials & experimental systems

n/a	Involved in the study
☒	☐ Antibodies
☒	☐ Eukaryotic cell lines
☒	☐ Palaeontology and archaeology
☒	☐ Animals and other organisms
☐	☒ Clinical data
☒	☐ Dual use research of concern
☒	☐ Plants

Methods

n/a	Involved in the study
☒	☐ ChIP-seq
☒	☐ Flow cytometry
☒	☐ MRI-based neuroimaging

Clinical data

Policy information about clinical studies

All manuscripts should comply with the ICMJE guidelines for publication of clinical research and a completed CONSORT checklist must be included with all submissions.

Clinical trial registration | Clinicaltrials.gov: NCT05684055

Study protocol | Full protocol available on clinicaltrials.gov; published protocol available open access: https://trialsjournal.biomedcentral.com/articles/10.1186/s13063-024-08226-2

Data collection | Data were collected by trained CHWs during home visits and entered into a mobile clinical decision support and data collection application. Enrolment lasted from September 08, 2023 to January 31, 2024.

Outcomes | The primary outcome was blood pressure control rate (<140/90 mmHg) twelve months after enrolment. Secondary endpoints included engagement in care, mean systolic and diastolic blood pressure at six and twelve months. Safety outcomes included deaths, non-fatal serious adverse events and adverse events of special interest, defined as events consistent with complications of hypertension or side-effects to antihypertensive medication. Endpoints were assessed by local Community Health Workers with the support via a dedicated clinical decision support and data collection application during home visits.

Plants

Seed stocks | *Report on the source of all seed stocks or other plant material used. If applicable, state the seed stock centre and catalogue number. If plant specimens were collected from the field, describe the collection location, date and sampling procedures.*

Novel plant genotypes | *Describe the methods by which all novel plant genotypes were produced. This includes those generated by transgenic approaches, gene editing, chemical/radiation-based mutagenesis and hybridization. For transgenic lines, describe the transformation method, the number of independent lines analyzed and the generation upon which experiments were performed. For gene-edited lines, describe the editor used, the endogenous sequence targeted for editing, the targeting guide RNA sequence (if applicable) and how the editor was applied.*

Authentication | *Describe any authentication procedures for each seed stock used or novel genotype generated. Describe any experiments used to assess the effect of a mutation and, where applicable, how potential secondary effects (e.g. second site T-DNA insertions, mosiacism, off-target gene editing) were examined.*
