# Supplementary material for: Lay community health worker-led care with mobile decision support for uncontrolled hypertension: a cluster-randomized trial
Source: Nat Med. 2026 Feb 12;32(3):915–23. doi: 10.1038/s41591-026-04208-w (PMC13768179; doi:10.1038/s41591-026-04208-w)
Supplement: Supplementary file 3 [file 41591_2026_4208_MOESM3_ESM.pdf]

nature portfolio

Peer Review File

Lay Community Health Worker-led versus professional facility-based hypertension care for people with uncontrolled blood pressure in rural Lesotho: a cluster-randomized trial within the ComBaCaL cohort study

Corresponding Author: Professor Niklaus Labhardt

Version 2:

Reviewer comments:

Reviewer #1

(Remarks to the Author)
The authors are to be commended for executing a well-designed and very informative study.
Strengths include the fact that it was embedded within the existing health system, the fact that CHWs were allowed to independently prescribe medications for controlling hypertension, and the use of open source software for the clinical decision support system. The findings here have the potential to influence policy in Lesotho and similar settings in LMICs
Comments:
Authorship-While this has nothing to do with the quality of the work, it is a bit disconcerting to see that out of 11 institutions represented in this article, 9 are foreign and only 2 are from Lesotho. This raises concerns about the real force driving this work and whether it will be sustainable.
Background
This is very well written and lays out a strong justification for conducting the study.
Methods
Procedures subheading should come before the outcomes subsection
L-577: Please specify what the definition of insufficient BP control was.
Excuse my ignorance, but it might be helpful to define what a village is in Lesotho. From Table 1, I see that the average village had 6 participants which seems rather small?
Sample size:
Could the authors please provide more details about the decision to recruit significantly more participants than what had been planned? Specifically when was this decision made, was it during data collection having reviewed accrued data or was it while blinded to the results of the study?
L651- a pilot study was conducted prior to the full study. What measures were taken in analysis to account for the fact that the areas where the pilot had been done had already been exposed to the intervention?
Results
The methods state that 103 villages were to be included but Table 1 has 97. Please explain the discrepancy
Discussion
L229- this is misleading. The relevant data to be used is the between group difference in BP change and not the absolute reduction in one arm
L249- It would be useful to further discuss what the effects of providinghte stipend to the CHWs was and how this can be mitigated to ensure sustainability of the intervention

(Remarks on code availability)

Reviewer #2

(Remarks to the Author)
Key Results: The results are clear and well displayed. Maybe consider the following suggestions:
It would be good to present flow chart of clusters and individuals from recruitment, randomization to final data collection stage

It would be good to report the characteristics for control and intervention group separately.
Table 1
Should have used a statistical test to test if there is any significant difference in the characteristics between the two study arms.
I suggest you report secondary endpoint at six month first, then the secondary endpoint at 12 months

Validity: The study was investigating a very important phenomenon and the results presented are interesting and significant in the discipline of health, particularly in chronic care management.
Data and methodology: There is no information on the methodology within the manuscripts except it being included in separately submitted document. Maybe it would be good to include the summary within the manuscript

Appropriate use of statistics and treatment of uncertainties: Statistics used were appropriate. For table 1, maybe consider using statistical test to test of there is any significance difference in the characteristics between the two study arms.

Conclusions: The data and results interpretations were robust and valid. However, in the discussion, very minimal was said about the results yet thats where they were supposed to be explicitly stated before being compared to existing literature.

Suggested improvement: Include a summary of the methodology within the manuscript

References: Well-articulated

Clarity and Context: Abstract well written. May consider the following suggestions:
Abstract: Rationale for conducting the cluster randomized trial has not been clearly stated; and summary of the objectives (both primary and secondary) were not stated
Introduction: You gave us information on poor access to hypertension care and initiatives done globally and in LMIC, but you didn't give information on the situation in Lesotho regarding the same phenomenon where this study took place. This information will help substantiate the problem that this study is trying to address, specifically in Lesotho;
Line 69-71: whats the magnitude of this insufficient access to the different hypertension services/care (preventive, diagnostic and therapeutic). Please highlight the magnitude of poor access and effects (ie mortality) that could be attributed to poor access to treatment. This information will help substantiate the problem that this study is trying to address.

(Remarks on code availability)

Reviewer #3

(Remarks to the Author)
The authors conducted a 1:1 cluster-randomized trial nested within the ComBaCaL cohort. They conclude that CHW-led hypertension care is safe and leads to better engagement in care and improved blood-pressure control versus facility-based management. Generally, this is a well-designed and well-reported cluster-randomized trial. The statistical methods are generally appropriate and clearly described in both the manuscript and the detailed SAP. The conclusions are supported by the data presented. Please see the points below that could be strengthened or warrant closer examination for the manuscript:

1. Please state clearly the primary outcome (BP control rates) in both the abstract and the main results section. The current manuscript makes it unclear for readers unless they read through the methodology sections.

2. The observed intra-cluster correlation coefficients (ICCs) for the endpoints are not reported. Please provide.

3. For some analyses, the authors describe a "missing = uncontrolled" rule for imputing absent endpoints, which is a conservative missing-data approach but not a traditional modified-ITT (mITT). Conventionally, mITT excludes participants who never initiate the intervention, whereas here all randomized participants remain in the analysis and only missing outcomes are imputed as uncontrolled. Moreover, it seems that the SAP does not define or reference a mITT population. Please clarify this terminology and ensure it aligns with the planned analysis.

4. Although age, sex, district, and access strata were adjusted, baseline SBP and DBP were not included in the primary regression model. Given the considerable variation in baseline BP, please explain why these measures were omitted and consider whether their inclusion might improve precision.

5. As the authors show, the number of participants per cluster varies from a median of 5-6. Are there very small clusters (especially single-participant clusters) that contribute little information but still enter the random effects? If there are, a sensitivity check excluding clusters below a minimal size could assess whether results hinge on very small clusters. As this could potentially destabilizing variance estimates, I would suggest the authors provide more detailed descriptions for the distribution of number of participants per cluster.

6. Mixed-effects models assume normally distributed random effects and linear relationships for continuous covariates on the logit scale. Please report diagnostic checks (e.g., residual plots, leverage or influence statistics) to demonstrate that these assumptions hold.

7. For Table 3: please include the absolute number of participants in each arm for every analysis (mITT and complete-case). Currently, Table 3's "engagement in care" rows list identical counts but differing proportions, which obscures the fact that denominators differ between mITT and complete-case analyses.

8. It is possible that the self-reported binary endpoint "engaged in care" mixes very different participant behaviors (some on optimal regimens, some just receiving any contact), potentially overstating clinical impact. Look at Table 3 for the " Adherence to antihypertensive st, n (%)" row: The adherence to medication appears lower in the intervention arm (77.9%) than the control arm (87.7%) at 12 months, although the difference is not statistically significant. This seems counterintuitive given the better BP control. It might be helpful to briefly comment on this finding in the discussion. I think it could be related to the definition of adherence, the populations being compared, or other factors. Do the authors have more granular data (e.g., number of CHW vs facility visits, medication adherence levels) that would clarify what "engagement" truly means for outcomes?

9. The authors should ensure they comply with both the CONSORT extension for cluster-randomized trials and the general CONSORT 2025 guidelines. The extension file has not yet been updated to reflect the 2025 version but please check.

10. Please clearly state the meaning of the intervals for Figure 2.

(Remarks on code availability)
The codes for the application of data collection and processing are provided, but it seems like codes for statistical analyses are not available now

Version 3:

Reviewer comments:

Reviewer #1

(Remarks to the Author)
I am happy with the responses and revisions that the authors have made in response to the comments that I made during the previous round of review. I have no further comments.

(Remarks on code availability)

Reviewer #2

(Remarks to the Author)
Comments have been comprehensively addressed

(Remarks on code availability)

Reviewer #3

(Remarks to the Author)
I have reviewed the responses to all my comments, and my concerns have been satisfactorily addressed in this version. I thank the authors for their clear and thorough responses

(Remarks on code availability)

Open Access This Peer Review File is licensed under a Creative Commons Attribution 4.0 International License, which permits use, sharing, adaptation, distribution and reproduction in any medium or format, as long as you give appropriate credit to the original author(s) and the source, provide a link to the Creative Commons license, and indicate if changes were made.
In cases where reviewers are anonymous, credit should be given to 'Anonymous Referee' and the source.
The images or other third party material in this Peer Review File are included in the article's Creative Commons license, unless indicated otherwise in a credit line to the material. If material is not included in the article's Creative Commons license and your intended use is not permitted by statutory regulation or exceeds the permitted use, you will need to obtain permission directly from the copyright holder.
To view a copy of this license, visit https://creativecommons.org/licenses/by/4.0/

To
Senior Editor Ming Yang and Reviewers
Nature Medicine

Basel, 29.08.2025

RE: Revision of the article "Community Health Worker-led versus facility-based hypertension care for people with uncontrolled blood pressure in rural Lesotho: a cluster-randomized trial within the ComBaCaL cohort study"; NMED-A140351A

Dear Dr. Yang, dear Reviewers

We would like to thank you for the careful review of our manuscript and the constructive comments. We feel that the comments helped to improve clarity of our manuscript.

Please find the point-by-point responses below

Yours sincerely

Prof. Niklaus Labhardt
Division and Department Head
Division of Clinical Epidemiology
Department of Clinical Research
University Hospital Basel

Comments Reviewer 1:

Number	Reviewer comment	Response	Manuscript changes
**1**	The authors are to be commended for executing a well-designed and very informative study.<br>Strengths include the fact that it was embedded within the existing health system, the fact that CHWs were allowed to independently prescribe medications for controlling hypertension, and the use of open source software for the clinical decision support system. The findings here have the potential to influence policy in Lesotho and similar settings in LMICs	Thank you for the appreciative comment.	None
**2**	**Authorship:** While this has nothing to do with the quality of the work, it is a bit disconcerting to see that out of 11 institutions represented in this article, 9 are foreign and only 2 are from Lesotho. This raises concerns about the real force driving this work and whether it will be sustainable.	Thank you for this comment. We agree that the aim must be to have more national institutions driving such publications. Our consortium is strongly committed at strengthening academic capacity in Lesotho. For example, there are 3 Basotho who are about to complete a PhD as part of the overall ComBaCaL project. While the majority of institutions represented are from outside Lesotho, the majority of authors are from the two institutions within the country. , When it comes to driving policy change and sustainability of projects, this is with the Ministry of Health of Lesotho, while the National University of Lesotho does not offer post-graduate courses in health science. Thus, the Basotho co-authors on this paper are from a local non-governmental organization and the Ministry of Health.	None

**3**	**Background:** This is very well written and lays out a strong justification for conducting the study.	Thank you	None
**4**	**Methods:** Procedures subheading should come before the outcomes subsection		Changed accordingly
**5**	L-577: Please specify what the definition of insufficient BP control was.	Thank you, we clarified in the sentence and replaced "insufficient" by the actual threshold.	**Methods**<br>*"Continued BP values ≥140/90 mmHg under high-dose antihypertensive fixed-dose combination, potential contraindications, side effects or the presence of clinical alarm signs or symptoms, triggered the CDSS application to recommend referral to the health facility."*
**6**	Excuse my ignorance, but it might be helpful to define what a village is in Lesotho. From Table 1, I see that the average village had 6 participants which seems rather small?	We added the information on the median eligible population size of villages into table 1	Median village eligible population size is now reported under Cluster-level characteristics in table 1.
**7**	Sample size: Could the authors please provide more details about the decision to recruit significantly more participants than what had been planned? Specifically when was this decision made, was it during data collection having reviewed accrued data or was it while blinded to the results of the study?	The sample-size calculation was made during study protocol development and thus **prior** to data collection. As stipulated in the study protocol, we decided prior to enrolment to include all participants with hypertension within the ComBaCaL cohort, even if it would exceed the calculated required minimum sample size. We added this information to the methods.	**Methods (section on sample-size):**<br>*"Given that the ComBaCaL cohort includes 103 villages with an expected 8 persons with uncontrolled hypertension per village, we anticipated to exceed the minimum sample-size of 304 participants."*
**8**	L651- a pilot study was conducted prior to the full study. What measures were taken in analysis to account for the fact that the areas where the pilot had been done had already been exposed to the intervention?	The pilot study was conducted in 10 villages that were **not** part of the ComBaCaL cohort. Thus, they were not already exposed to the intervention.	**Methods:**<br>*"Prior to study start, the intervention was piloted in ten villages outside the ComBaCaL cohort in both study districts."*

**9**	**Results**: The methods state that 103 villages were to be included but Table 1 has 97. Please explain the discrepancy	In six villages no eligible participants were found (no ComBaCaL cohort participants with uncontrolled hypertension)	**Results, first paragraph:** *"They identified 1'262 cohort participants with hypertension, out of which 547 (274 control, intervention) from 97 villages (47 intervention, 50 control) had uncontrolled BP levels (≥140/90mmHg) and were not pregnant (figure 1). In six villages no cohort participants were eligible for this trial"*
**10**	**Discussion:** L229- this is misleading. The relevant data to be used is the between group difference in BP change and not the absolute reduction in one arm	We agree and deleted this sentence	Sentence deleted
**11**	L249- It would be useful to further discuss what the effects of providing the stipend to the CHWs was and how this can be mitigated to ensure sustainability of the intervention	We thank the reviewer for this important point. All CHWs in both arms received the same non–performance-based stipend to compensate for additional study-related workload. As such, it cannot explain between-group differences. We agree that sustainable CHW compensation is essential, and have added a concise statement in the discussion highlighting the need for adequate support structures and future cost-effectiveness assessments.	**Discussion** *All CHWs in both arms received the same non–performance-based stipend, which compensated study-related workload and cannot explain between-group differences. Sustainable scale-up will require realistic workload, adequate support structures, and cost-effectiveness assessments to inform appropriate CHW compensation.*

Comments Reviewer 2:

Number	Reviewer comment	Response	Manuscript changes
**1**	Key Results: The results are clear and well displayed. Maybe consider the following suggestions: It would be good to present flow chart of clusters and individuals from recruitment, randomization to final data collection stage.	We added the number of clusters at each stage in the revised figure 1	Revised figure 1
**2**	It would be good to report the characteristics for control and intervention group separately.	We provide participant characteristics stratified by group in table 1	Tabe 1 (unchanged)
**3**	Table1: Should have used a statistical test to test if there is any significant difference in the characteristics between the two study arms.	We appreciate the reviewer's suggestion. However, formal hypothesis testing for differences in baseline characteristics in randomized trials is no longer recommended in contemporary statistical guidance. The purpose of randomization is to ensure that treatment groups are comparable *on average* with respect to both measured and unmeasured characteristics; any observed baseline imbalances are the result of chance rather than systematic bias. Significance testing in this context can be misleading, as the *p*-values reflect sample size and random variation rather than the success of randomization, and they can encourage inappropriate post-hoc adjustments[1].<br>Instead, current CONSORT recommendations and related methodological literature advise presenting descriptive summaries (e.g., means, standard deviations, proportions) without inferential tests, to allow readers to judge the clinical relevance of any observed differences[2].<br>We have therefore kept Table 1 as a purely descriptive table, in line with these guidelines.	None
**4**	I suggest you report secondary endpoint at six months first, then the secondary endpoint at 12 months	Thank you for this comment. We believe that it is more important to report the primary endpoint first rather than following the chronological order. We	None

		hope that this is acceptable to the reviewing team. .	
**5**	Validity: The study was investigating a very important phenomenon and the results presented are interesting and significant in the discipline of health, particularly in chronic care management.	Thank you very much for this comment.	None
**6**	Data and methodology: There is no information on the methodology within the manuscripts except it being included in separately submitted document. Maybe it would be good to include the summary within the manuscript.	This request has been discussed with the handling editor. Due to editorial formatting choices, we are not allowed to include methodological information within the main manuscripts.	None
**7**	Appropriate use of statistics and treatment of uncertainties: Statistics used were appropriate. For table 1, maybe consider using statistical test to test of there is any significance difference in the characteristics between the two study arms.	Kindly refer to our response to question 3 from reviewer 2.	None
**8**	Conclusions: The data and results interpretations were robust and valid. However, in the discussion, very minimal was said about the results yet that where they were supposed to be explicitly stated before being compared to existing literature.	Thank you for this suggestion. We added a first paragraph.	**Discussion (Paragraph 1):**<br>*"In this cluster-randomized trial in rural Lesotho, CHWs who independently initiated, dispensed, and titrated antihypertensive medication with the support of a CDSS achieved substantially higher rates of BP control at 12 months than CHWs restricted to screening and referral for facility-based care. The intervention also improved engagement in care and linkage to treatment, without safety concerns. In Lesotho, CHWs are lay community members with limited formal training, accredited and supported by the Ministry of Health. To our knowledge, this is the first trial to demonstrate that such a cadre can safely and effectively deliver hypertension care when supported by a CDSS. These findings highlight the potential of task-shifting models involving minimally trained CHWs in similar low-income settings."*

**9**	Suggested improvement: Include a summary of the methodology within the manuscript.	This request has been discussed with the handling editor. Due to editorial formatting choices, we are not allowed to include methodological information within the main manuscripts.	None.
**10**	References: Well-articulated	Thank you.	None
**11**	Clarity and Context: Abstract well written. May consider the following suggestions:<br>Abstract: Rationale for conducting the cluster randomized trial has not been clearly stated; and summary of the objectives (both primary and secondary) were not stated	Thank you for this comment. Based on this comment and the editor's comment number 9, we completely revised the abstract.	Revised abstract.
**12**	Introduction: You gave us information on poor access to hypertension care and initiatives done globally and in LMIC, but you didn't give information on the situation in Lesotho regarding the same phenomenon where this study took place. This information will help substantiate the problem that this study is trying to address, specifically in Lesotho;<br>Line 69-71: whats the magnitude of this insufficient access to the different hypertension services/care (preventive, diagnostic and therapeutic). Please highlight the magnitude of poor access and effects (ie mortality) that could be attributed to poor access to treatment. This information will help substantiate the problem that this study is trying to address.	Thank you for this comment, we added information on the situation in Lesotho, building on three studies recently published.	Introduction:<br>*" In Lesotho, a predominantly rural low-income country in southern Africa, a recent population-based survey found a hypertension prevalence of 21.6% among adults aged ≥18 years[3]. Among those affected, 70% were aware of their condition, 67% reported taking treatment, yet only 49% achieved BP control (<140/90 mmHg)[4], and a considerable proportion already showed signs of end-organ damage such as retinopathy and nephropathy[5]. This growing burden of hypertension adds to Lesotho's high prevalence of HIV and tuberculosis, reflecting the double burden of infectious and non-communicable diseases[6]. Faced with this challenge, and given the constraints of a weak health system and shrinking international funding for chronic care, there is an urgent need for innovative and affordable models of service delivery.»*

Comments Reviewer 3:

Number	Reviewer comment	Response	Manuscript changes
**1**	The authors conducted a 1:1 cluster-randomized trial nested within the ComBaCaL cohort. They conclude that CHW-led hypertension care is safe and leads to better engagement in care and improved blood-pressure control versus facility-based management. Generally, this is a well-designed and well-reported cluster-randomized trial. The statistical methods are generally appropriate and clearly described in both the manuscript and the detailed SAP. The conclusions are supported by the data presented. Please see the points below that could be strengthened or warrant closer examination for the manuscript:	Thank you.	None
**2**	Please state clearly the primary outcome (BP control rates) in both the abstract and the main results section. The current manuscript makes it unclear for readers unless they read through the methodology sections.	Thank you, the abstract was fully revised and the primary outcome is clearly stated.	**Abstract**:<br>*"The primary objective was to assess the effectiveness and safety of CHW-led care, with the primary outcome defined as BP <140/90 mmHg at 12 months..»*<br><br>**Results (primary outcome):**<br>*«For the primary outcome of blood pressure control (<140/90 mmHg) at 12 months, the modified intention-to-treat analysis included 543 participants (four were excluded due to pregnancy during follow-up). BP control was achieved by 156 of 271 participants (58%) in the intervention arm and 130 of 272 participants (48%) in the control arm (Table 2)."*
**3**	The observed intra-cluster correlation coefficients (ICCs) for the endpoints are not reported. Please provide.	Based on comment 10 of the reviewer 3, we added for all outcomes in table 2 the ICC as footnote.	**Results:**<br>Footnotes in table 2
**4**	For some analyses, the authors describe a "missing = uncontrolled" rule for imputing absent endpoints, which is a conservative missing-data approach but not a traditional modified-ITT (mITT). Conventionally, mITT excludes participants who never initiate the intervention, whereas here	Thank you for this comment. We agree. Actually in the SAP we label it as ITT (and not as mITT). We have thus changed from	Changed from mITT to ITT throughout the manuscript and the methods.

	all randomized participants remain in the analysis and only missing outcomes are imputed as uncontrolled. Moreover, it seems that the SAP does not define or reference a mITT population. Please clarify this terminology and ensure it aligns with the planned analysis.	mITT to ITT throughout the manuscript.	
**5**	Although age, sex, district, and access strata were adjusted, baseline SBP and DBP were not included in the primary regression model. Given the considerable variation in baseline BP, please explain why these measures were omitted and consider whether their inclusion might improve precision.	Controlled BP (yes/no) was analyzed in the primary analysis without adjustment for baseline status as all participants are uncontrolled. As displayed in Table 1, systolic and diastolic blood pressures were well balanced across study arms with a mean (sd) systolic BP of 147.5 (17.0) and 150.5(16.5) in control and intervention, respectively. Mean (sd) diastolic BP were 94.5 (9.3) and 95.4 (10.9) in control and intervention, respectively.<br>Controlling for baseline systolic and diastolic blood pressure would give robust interpretation of our trial with an estimated effect of intervention of 1.56 (95%CI 1.03-2.35) compared to the reported effect (not adjusted for systolic and diastolic BP) ot 1.52 (1.01-2.29). For the	None

		sake of not fishing for results, we propose to stick to the analysis pre-specified in our protocol.	
**6**	As the authors show, the number of participants per cluster varies from a median of 5-6. Are there very small clusters (especially single-participant clusters) that contribute little information but still enter the random effects? If there are, a sensitivity check excluding clusters below a minimal size could assess whether results hinge on very small clusters. As this could potentially destabilizing variance estimates, I would suggest the authors provide more detailed descriptions for the distribution of number of participants per cluster.	We did not pre-specify a minimum size per cluster as we believed that small clusters could still contribute to the estimation of the fixed effects. However, the mixed cluster effect was removed from secondary analysis models when we faced singularity issues. Hence, we drop the random effect rather than dropping clusters, according to singularity/convergence diagnostics.	Methods (statistical analysis):<br>"Whenever a variance component of the mixed effects was estimated as negligeable and led to singularity diagnostics, we simplified the model by omitting the corresponding random effect rather than excluding clusters from the analysis"
**7**	Mixed-effects models assume normally distributed random effects and linear relationships for continuous covariates on the logit scale. Please report diagnostic checks (e.g., residual plots, leverage or influence statistics) to demonstrate that these assumptions hold.	We thank the reviewer for this suggestion. Diagnostic checks showed no severe violations of model assumptions. Random effects were approximately normally distributed (QQ-plots) and the continuous covariate age showed a linear relationship with the outcome on the logit scale (binned residual plots). We did not observe influential clusters or outliers affecting model stability. We	Results section:<br>*Diagnostic checks indicated no major violations of model assumptions; random effects were approximately normally distributed, and the relationship of age with the outcome was linear on the logit scale.*

		have added a concise statement to the Results.	
**8**	For Table 3: please include the absolute number of participants in each arm for every analysis (mITT and complete-case). Currently, Table 3's "engagement in care" rows list identical counts but differing proportions, which obscures the fact that denominators differ between mITT and complete-case analyses.	Thank you for spotting this.	**Footnotes table 3:**<br>*« 4: Only including participants with available endpoint data at 12 months: 240 control, 227 intervention*<br>*9: Only including those with available endpoint data at 6 months: 250 control, 236 intervention »*<br>-
**9**	It is possible that the self-reported binary endpoint "engaged in care" mixes very different participant behaviors (some on optimal regimens, some just receiving any contact), potentially overstating clinical impact. Look at Table 3 for the " Adherence to antihypertensive st, n (%)" row: The adherence to medication appears lower in the intervention arm (77.9%) than the control arm (87.7%) at 12 months, although the difference is not statistically significant. This seems counterintuitive given the better BP control. It might be helpful to briefly comment on this finding in the discussion. I think it could be related to the definition of adherence, the populations being compared, or other factors. Do the authors have more granular data (e.g., number of CHW vs facility visits, medication adherence levels) that would clarify what "engagement" truly means for outcomes?	As stipulated in the methods section "Outcomes" and in the footnotes of table 3, engagement in care is defined as reporting current use of antihypertensive medication or reaching treatment targets without medication use. Adherence was assessed asking on how many of the last four days a dose of antihypertensive medication was missed. Participants reporting no dose missed over the last four days were considered adherent. We have clarified the corresponding table footnotes and added the definition of adherence in the methods section "Outcomes"	*Table 3 footnotes:*<br><br>*Engagement in care: "Reporting current use of antihypertensive medication or reaching treatment target without medication"*<br><br>*Adherence: "Reporting no dose of antihypertensive medication missed over the last four days."*<br><br>*Methods, Outcomes:*<br><br>*Adherence to antihypertensive treatment was assessed asking how many of the last four days a dose of antihypertensive medication was missed. Participants reporting no doses missed over the last four days were considered adherent.*
**10**	The authors should ensure they comply with both the CONSORT extension for cluster-randomized trials and the general CONSORT 2025 guidelines. The extension file	We thank the reviewer for this important comment. We carefully revised the manuscript for compliance	**Results:**<br>First paragraph: *"All 103 villages in the ComBaCaL cohort were included and randomized; no clusters declined participation or were excluded. "*

	has not yet been updated to reflect the 2025 version but please check.	with CONSORT 2025 and the cluster RCT extension. Specifically, we now (i) added explicit reporting of cluster inclusion at the start of the Results, (ii) provided cluster size distribution, (iii) reported observed intra-cluster correlation coefficients (ICCs) for the primary and key secondary outcomes in table footnotes, and (iv) strengthened external validity considerations in the Discussion.	- Added range of participants per cluster in table 1<br>- Added ICC for all outcomes reported in table 2 as footnotes<br><br>**Discussion:**<br>"*The findings of our trial are likely to be generalizable to other rural low-income where a CHW system is already in place or being rolled out. However, generalizability may be more limited in urban contexts or in health systems with different task-shifting regulations."*
**11**	Please clearly state the meaning of the intervals for Figure 2.	Thank you, we expanded our figure legend accordingly	Legend of Figure 2:<br><br>*"Statistics are derived using marginal standardization. Confidence intervals reflect the 2.5th–97.5th percentiles from 1000 bootstrap samples"*
**12**	The codes for the application of data collection and processing are provided, but it seems like codes for statistical analyses are not available now.	We uploaded the statistical code on the Zenodo repository	Code availability statement<br>New doi provided for updated version of the zenodo repository containing the statistical code alongside the data set.

Comments Editor:

Number	Editor's comment	Response	Manuscript changes
**1**	The peer reviewers find the study is of high interest and important. However, they have some concerns particularly around the conceptualisation of the study to Lesotho context well as the potential real-world and community health impact of this study. In this case, it would be very important for you to address the following editorial points (please address them in your point-by-point response letter):		
**2**	Please provide more context into background of hypertension burden and current state of healthcare systems in Lesotho context. This will inform the rationale for the study.	Considering this comment and the comment 12 of reviewer 2, we expanded the introduction.	**Introduction**:<br>*"In Lesotho, a predominantly rural low-income country in southern Africa, a recent population-based survey found a hypertension prevalence of 21.6% among adults aged ≥18 years[3]. Among those affected, 70% were aware of their condition, 67% reported taking treatment, yet only 49% achieved BP control (<140/90 mmHg)[4], and a considerable proportion already showed signs of end-organ damage such as retinopathy and nephropathy[5]. This growing burden of hypertension adds to Lesotho's high prevalence of HIV and tuberculosis, reflecting the double burden of infectious and non-communicable diseases[6]. Faced with this challenge, and given the constraints of a weak health system and shrinking international funding for chronic care, there is an urgent need for innovative and affordable models of service delivery.»*
**3**	Please provide more information on the real-world impact of the intervention to the community, particularly in the discussion.	We agree and have expanded the Discussion to highlight the real-world feasibility and impact of the intervention. We emphasize that CHWs successfully and independently delivered hypertension care at the village level, thereby reducing reliance on health facilities and improving access in remote	**We added to the Discussion:**<br>Beyond its clinical effectiveness, the intervention demonstrated real-world feasibility: CHWs successfully and independently delivered guideline-based hypertension care at the village level, thereby reducing reliance on health facilities and expanding access to treatment in remote rural areas.

		rural areas. These changes were made in the main manuscript Discussion.	
**4**	Please provide more information on how community participation has been involved in the co-creation of the study, as well as how policymakers were involved.	We thank the editor for this comment. We revised the Methods to clarify the role of CHWs in both arms and moved the description of study procedures above outcomes for improved clarity.	**Methods**<br>*“The trial was designed and implemented in partnership with the Lesotho Ministry of Health, who acted as co-investigators, and with input from community leaders in participating villages. Local CHWs were centrally involved in planning and delivery, ensuring feasibility and cultural appropriateness.»*<br><br>**Discussion:**<br>“*The study was co-developed with the Lesotho Ministry of Health, with active engagement of community leaders and CHWs, which strengthened feasibility, cultural appropriateness, and alignment with national health priorities. These features enhance the relevance of our findings and support their potential scalability in similar low-income rural settings.”*
**5**	It seems the lay health worker delivery model is a unique aspect of the study, and this needs to be emphasized more. Please discuss how lay health workers are different from standard community health workers, as well as the impact of such health worker system integrated into the context of Lesotho health system.	We thank the editor for this important point. In Lesotho, CHWs are lay volunteers elected by their communities, accredited by the Ministry of Health, and receive a small stipend. They mainly provide health promotion and linkage to facility-based care, particularly for maternal and child health and HIV, and thus differ from more medically trained cadres such as “village doctors” in China. To clarify this, we revised the “Study design and setting” section to explicitly describe the CHW cadre in Lesotho. In the Discussion, we highlight that demonstrating the effectiveness of such minimally trained, government-accredited CHWs in delivering hypertension care adds to the evidence for	**Methods (design and setting):**<br>*CHWs play an important role in the Lesotho healthcare system, providing health promotion services and linking the community to facility-based care, mainly for maternal and child health, tuberculosis and HIV. In Lesotho, CHWs are lay volunteers elected by their communities, accredited by the Ministry of Health, and receive a monthly stipend of 800 Lesotho Maloti (approximately 40 USD)3–5.*<br><br>Discussion (first paragraph)<br>*… In Lesotho, CHWs are lay community members with limited formal training, accredited and supported by the Ministry of Health. To our knowledge, this is the first trial to demonstrate that such a cadre can*

		scalable task-shifting models in similar LMIC settings.	*safely and effectively deliver hypertension care when supported by a CDSS. These findings highlight the potential of task-shifting models involving minimally trained CHWs in similar low-income settings.*<br><br>Further down in the discussion we emphasize again the difference between CHWs in most LMICs and the ones in China:<br>*"…However, these findings [referring to the findings from China] may not be generalizable to an African context[24]. CHWs in China, called "village doctors", are accredited after three years of medical education or twenty years of continuous practicing in village clinics and have routine prescription rights for essential medications, unlike CHWs in most LMICs[34]."*
**6**	Similarly, please provide more information on the mobile decision support tool, what it entails, how it functions from other telemedicine platforms and how it is used by lay health workers.	Thank you for this comment. It is true that the mobile clinical decision support tool was a key component of the intervention. We have published two manuscripts on the pilot version of the tool with further information[9,10] and plan to publish another separate publication on the usability of the tool in the context of chronic care delivery by CHWs. For the current manuscript a full elaboration on the functionalities and usability of the tool is out of scope, but we have added some further details in the Methods section. The code for the application is freely available on github under the link provided in the Code availability statement.	**Methods, Procedures:**<br>*The CDSS application provided algorithmic guidance for all study procedures, clinical procedures, and served as a data collection tool. The application was configured by the study team based on the open-source Community Health Toolkit* (CHT) *Core Framework (versions 3.12, 3.14, 4.6 and 4.13), a widely-used, open source software toolkit designed for community health systems[10]. As a progressive web app with an offline-first approach, CHT is particularly well-suited for settings with limited internet connectivity. It provides a core framework and configuration code that enable the rapid development and deployment of scalable digital health applications on Android-based mobile devices[10]. The application allowed CHWs to register households and clients, document electronic written consent, and guided them via automated task scheduling*

			and structured questionnaires through all clinical procedures and data collection. Furthermore, the application provided individual client overviews and an aggregated data overview with target metrics.
**7**	Nature portfolio journals have introduced a new approach to improving inclusion in authorship representation (please see the recent editorial here https://www.nature.com/articles/d41586-022-01423-6). Please include an "Ethics and Inclusion statement" in the methods section. Please see our editorial policies on inclusion and ethics in global research here: https://www.nature.com/nature-portfolio/editorial-policies/authorship#authorship-inclusion-and-ethics-in-global-research. Please see a recent example of an ethics and inclusion statement here: https://www.nature.com/articles/s41591-023-02288-6#Sec12.	Already addressed in first submission	None
**8**	Editorial points to be addressed. Please note that not all of these might apply to your manuscript, in which case please respond with “not applicable.” Structural changes to re-organise the manuscript do not need to be tracked.	Noted	None
**9**	Abstract should be ~200 words and for trials should adhere to the CONSORT framework. You must state if the primary outcome was or was not met and provide the effect size and relevant uncertainty estimate. The conclusion of the study must focus only on the primary outcome and safety/tolerability. You must report either all or none of the secondary outcomes, given the space limitation, I would suggest removing all mention of secondary outcomes from the abstract.	We have rewritten the abstract to comply with all aspects mentioned by the editor and the reviewers.	See revised abstract
**10**	Please include the trial registration number at the end of the Abstract.	Already addressed in first submission	None
**11**	The Introduction should be written for a broad, non-specialist medical reader and provide sufficient context for the work.	We expanded on the context based on your comment number 2	See editor comment 2
**12**	Please provide details of your cohort in the first paragraph of the Results. This includes number of individuals screened for enrolment, as well as exact dates	The number of cohort participants eligible for screening, the number screened and the exact	None

	of first and last patient enrolment. The CONSORT patient disposition diagram and the baseline characteristics must be included as main non-text items (for which there is a strict limit of 6 tables and/or figures).	date of first and last enrolment are provided in the first paragraph of the Results section. The CONSORT diagram is provided in figure 1 and baseline characteristics in table 1.	
**13**	The Results should be structured as followed:<br>- Patient disposition<br>- Primary outcome(s)<br>- Secondary outcomes<br>- Safety<br>- Exploratory outcomes<br>- Sensitivity analyses<br>- Post-hoc analyses	We revised the order in the results section and introduced subtitles to reflect the structure.	See revised results section.
**14**	Please remove any subheadings from the Discussion.	Already addressed in first submission	None
**15**	Please ensure all results are presented in the Results section, no new data should be introduced in the Discussion.	Already addressed in first submission	None
**16**	You must include explicit paragraphs of study limitations in the Discussion.	Already addressed in first submission	None
**17**	The overarching conclusion of the study must be based only on the primary outcome and safety data.	Addition of a last conclusion paragraph in the Discussion section	**Discussion (last paragraph):**<br>*"This cluster-randomized trial demonstrates that CHW-led hypertension care, supported by a mobile CDSS, improved blood pressure control at 12 months without safety con18cerns. Health policies should consider empowering CHWs to independently initiate and titrate antihypertensive treatment for uncomplicated hypertension to address prevailing gaps in hypertension care."*
**18**	The Methods should include a full description of the inclusion and exclusion criteria, as well as study procedures and statistical analyses (including a power calculation).	Already addressed in first submission	None
**19**	Please upload the protocol and SAP with the revision materials, so that reviewers and editors have access to them.	SAP already uploaded with first submission and available on clinicaltrials.gov.	None
**20**	You must ensure that contributions from all individuals in the author list are available in the Author Contributions statement.	Checked	Added missing author contributions.

**21**	Please move all funding sources to the Acknowledgements, including a statement on the role of the funder.	Already addressed in first submission	None
**22**	Please ensure that all potential competing interests are detailed for all authors. For any authors with no competing interests, this must also be stated.	Already addressed in first submission	None
**23**	Please see our guidelines for the Data and Code Availability Statements. "Available on request" is not acceptable, you must provide details of any restrictions to data and code availability. https://www.nature.com/nature-portfolio/editorial-policies/reporting-standards#availability-of-data.	A de-identified key dataset and the code for the CDSS application have been available since the initial submission with corresponding links to zenodo and github provided in the Data and Code availability statements. In addition, we have now added the statistical code for analysis on the zenodo repository and updated the statement accordingly, including an updated link to the new version of the repository.	*«A de-identified key dataset alongside the statistical code for reproducing the primary and secondary endpoints is available at https://doi.org/10.5281/zenodo.16903262."*
**24**	* The article file must only contain these items in this order:<br>• Title<br>• Author List and affiliations<br>• Abstract<br>• Introduction<br>• Results (with Subheadings)<br>• Discussion<br>• Acknowledgements<br>• Author Contributions<br>• Competing Interests Statement<br>• References (for main text only)<br>• Figure legends (for main text only)<br>• Tables (note: tables should be pasted into Word files as editable tables, not as images)<br>• Methods<br>• Data Availability Statement<br>• Code Availability Statement<br>• Methods-only References	Already addressed in first submission	None
**25**	Include a point-by-point response to all editorial queries below. For any points that do not apply to your revision, please state "not applicable."	Done	

**26**	Include a “Response to referees” document detailing, point-by-point, how you addressed each referee comment. If no action was taken to address a point, you must provide a compelling argument. This response will be sent back to the referees along with the revised manuscript.	Done	
**27**	If you have not already done so, please begin to revise your manuscript so that it conforms to our policy and format instructions here: https://www.nature.com/nm/for-authors/preparing-your-submission#formatting Refer also to any guidelines provided in this letter.<br>Submit both clean and tracked changes versions of the revision as editable Word documents.	Done	
**28**	Include a revised version of any required reporting checklist. The reporting summary can be found at https://www.nature.com/documents/nr-reporting-summary.pdf. Please note that this form is a dynamic ‘smart pdf’ that must be downloaded and completed in Adobe Reader (i.e. cannot be opened in a web browser).	Done	
**29**	If the research findings apply to only one sex or gender, that must be indicated in the title and/or abstract.	NA	
**30**	For studies involving vertebrates animal and cell lines- The Reporting Summary should include whether sex was were considered in the study design.	NA	
**31**	For studies involving human research participants- The Reporting Summary should include whether sex and/or gender was considered in the study design and whether sex and/or gender of participants was determined based on self-report or assigned (and methodology used).	Sex was considered in the study design as a baseline characteristic, but the trial was not stratified by sex. Sex of participants was self-reported at enrollment. The analysis adjusted for sex as a baseline covariate.	We specified that we collected self-reported sex under screening and enrolment of the Methods section:<br>*“At enrolment, CHWs collected baseline sociodemographic and clinical information, including self-reported sex, age, medical history, and blood pressure.”*<br>And specify it in the abstract:<br>*“Following community-based hypertension screening, 547 non-pregnant adults with blood pressure (BP) ≥140/90 mmHg were*

			enrolled (75% self-reported female sex; median age 64 years, mean BP 149/95 mmHg)»
**32**	Data should be reported disaggregated for sex and gender where this information has been collected and consent has been obtained for reporting and sharing individual-level data; disaggregated numbers for individual experiments must be provided in the source data as appropriate whereas overall numbers may be provided in the Nature Portfolio Reporting Summary.	The SAP and the protocol foresee sex as a fixed effect in the primary endpoint analysis. In a pre-specified analysis, the interaction of sex with the intervention effect was assessed. As there was no relevant interaction of sex with the intervention effect, the subgroup analysis by sex is only provided in the extended data file but not in the main manuscript.	In the results it is already reported: *"Interaction p-values for the pre-specified potential effect modifiers (sex, age, engagement in hypertension care at baseline, access to health facility, and engagement in HIV care) were larger than 0.1 and therefore no subgroup credibility assessments were performed (see extended data table 4 and extended data figure 1). "*
**33**	Information on the 3 points above should be included in the revised manuscript and detailed in the cover letter.	Done	
**34**	In addition, please note that if sex- and gender-based analyses have been performed a priori, results should be reported regardless of positive or negative outcome. We discourage conducting post hoc sex- and gender-based analysis if the study design is insufficient (for example, low sample size) to enable meaningful conclusions.	NA	
	If no sex- and gender-based analyses have been performed, please indicate the reasons for the lack of these analyses in the Reporting Summary.	The proportion of male and female in the study population is reported. In a pre-specified analysis the interaction of sex with the intervention effect was assessed. As there was no relevant interaction of sex with the intervention effect, the subgroup analysis by sex is only provided in the extended data file but not in the main manuscript.	
**35**	When submitting the revised version of your manuscript, please also pay close attention to our href="https://www.nature.com/nature-research/editorial-policies/image-integrity">Digital Image Integrity Guidelines. and to the following points below:	Done	
**36**	that unprocessed scans are clearly labelled and match the gels and western blots presented in figures.	NA	

**37**	that control panels for gels and western blots are appropriately described as loading on sample processing controls	NA	
**38**	all images in the paper are checked for duplication of panels and for splicing of gel lanes.	NA	
**39**	Finally, please ensure that you retain unprocessed data and metadata files after publication, ideally archiving data in perpetuity, as these may be requested during the peer review and production process or after publication if any issues arise.	Done	
**40**	Nature Medicine is committed to improving transparency in authorship. As part of our efforts in this direction, we are now requesting that all authors identified as 'corresponding author' on published papers create and link their Open Researcher and Contributor Identifier (ORCID) with their account on the Manuscript Tracking System (MTS), prior to acceptance. This applies to primary research papers only. ORCID helps the scientific community achieve unambiguous attribution of all scholarly contributions.	Done	

To
Senior Editor Ming Yang and Reviewers
Nature Medicine

Basel, 29.09.2025

RE: Revision of the article “Community Health Worker-led versus facility-based hypertension care for people with uncontrolled blood pressure in rural Lesotho: a cluster-randomized trial within the ComBaCaL cohort study”; NMED-A140351A

Dear Dr. Yang, dear Reviewers

We would like to thank you again for the careful review of our manuscript.

Please find the point-by-point responses to the remaining comments below

Yours sincerely

Prof. Niklaus Labhardt
Division and Department Head
Division of Clinical Epidemiology
Department of Clinical Research
University Hospital Basel

Comments Editor:

Number	Comment	Response	Manuscript changes
**1**	Your revised Article, "Community Health Worker-led versus facility-based hypertension care for people with uncontrolled blood pressure in rural Lesotho: a cluster-randomized trial within the ComBaCaL cohort study" has now been seen by 3 referees. You will see from their comments that they find your revised manuscript strengthened and improved. In light of their positive feedback, and provided that you can address their remaining concerns as well as our editorial points below, we would be happy to offer to publish your study in Nature Medicine once this next round of revision is completed.	Thank you for the appreciative comment. We hope that we addressed all the points satisfactorily and thank you for the opportunity to publish at NatureMedicine	None
**2**	Given the conceptual novelty of the trial in incorporating lay health workers and the use of the decision support tool, this needs to be emphasized throughout the manuscript. Therefore, please change from "community health workers" (which is a general and broad term) to "lay health workers" throughout the abstract and manuscript.	Thank you for this comment. We appreciate your point that the novelty and importance of our trial lies in demonstrating that it was conducted with **lay** CHWs operating within the routine health system. We have now consistently changed the wording to "lay CHW" throughout the manuscript. Only in instances where we discuss CHWs more broadly we omitted the qualifier "lay." In addition, we added "professional" to "facility-based care" in the control arm to better emphasize the contrast between lay-CHW-led and professional facility-led care in the trial where appropriate.	Changed to "lay CHW" throughout the manuscript.
**3**	It is also important to provide more context into why lay health workers are important and unique in the context of Lesotho (you mentioned elsewhere they are embedded into existing government-led community health programs). Please also provide more background into the nature of the decision support tool in the introduction- how does it work, and how does it aid lay health workers.	We have added to the introduction 3 sentences summarizing the current CHW-program in Lesotho to emphasize our trial's integration into the national system.	**Introduction:**<br>Addition of:<br>*"Lesotho has an established community health system in which lay persons, elected by their communities and accredited after a short train-*

		Additional information on the CDSS application has been added in the introduction as suggested.	*ing, serve part-time as CHWs and receive a stipend. Reflecting the country's mountainous geography with many small, remote villages, the national strategy targets a CHW-to-population ratio of 1:250[1]. The responsibilities of Lesotho's lay CHWs include health promotion and education, nutrition and growth monitoring, and the sensitization, screening, and referral of individuals for communicable diseases such as HIV and tuberculosis. As in most countries in the region, hypertension diagnosis and treatment are not yet part of CHWs' routine tasks in Lesotho."*<br><br>And:<br><br>*"CDSS directly assist clinical decision-making by providing personalized assessments and recommendations based on individual patient characteristics. By offering algorithmic guidance and facilitating remote supervision, mobile CDSS may enhance the quality, efficiency, and scope of CHW services."*
**4**	Please separate "Further secondary and exploratory outcomes" to secondary and exploratory outcomes sections, specifically.	We have changed this accordingly.	
**5**	You currently have 8 main display items (4 tables, 4 figures), and 5 extended display items. Please convert 2 of your main display items to extended display items to enhance visibility (each of which must fit on a single page), and/or merge some figures together (I suggest merging Figures 3 and 4 into a single Figure). Please note that we can permit 6 main display items and 10 Extended Data display items. Please convert some of your tables to Supplementary Tables, of which you can have an unlimited number.	We have reduced the number of display items to match the requirements. All items included as extended display items are a direct extension of findings reported in the main manuscript (i.e. additional baseline and outcome data that did not fit into the main tables). Therefore, we would suggest to keep them in the Extended Data file.	**Results**<br>Merged figure 3 and 4<br>Merged table 2 and 3

**6**	Please remove editorialising terms/language throughout the manuscript when describing the study and findings- i.e. "first", "novel", "largest/most comprehensive", "clearly", "successful"	Done	All editorializing terms have been removed throughout the manuscript
**7**	Please provide a separate section on "Ethics and Approval"- detailing informed consent etc. that is separate from the Inclusion and ethics statement	Done	**Ethics and approval**<br>*"The trial was approved by the National Health Research and Ethics Committee (NH-REC) of Lesotho (ID 102-2022). Additionally, the Ethikkomission Nordwest- und Zentralschweiz (EKNZ) in Switzerland provided a statement confirming the trial meets all ethical requirements for a Swiss research project (ID AO_2022_00074).*<br>*The trial was nested within the ComBaCaL cohort study following the Trials within Cohort (TwiCs) design. Following the French consent pattern of the TwiCs design, the consent for the ComBaCaL cohort included consent to being randomized as part of future nested trials. The consent form is available as supplementary file of the ComBaCaL cohort profile publication and on clinicaltrials.gov (NCT05596773).*<br>*Consent was obtained at three levels. First, oral consent from the village chiefs was obtained during village eligibility assessment prior to the CHW selection and enrolment of a village into the ComBaCaL cohort. Second, oral consent was obtained from the household head prior to the registration of a household in the ComBaCaL cohort data base and to approaching household members for individual consent, Third, individual written consent was obtained using a tablet-based consent application. Based on participants' characteristics (age, literacy, need for a guardian), the consent application displayed appropriate simplified study information and allowed for electronic signature on the informed consent form. Illiterate participants confirmed*

			informed consent by drawing a cross in the electronic signature field, countersigned by an impartial witness. Signed consent forms were uploaded in the study database of the CDSS application and checked for correctness and completeness by the study team remotely and re-consent tasks were triggered via the CDSS application in case of errors.<br>*Consent can be withdrawn any time on individual or household level. Pseudo-anonymized data collected until the time of withdrawal is retained in the database.*<br>*Following the TwiCs design, no trial-specific consent was required after identification of trial-eligible ComBaCaL cohort participants and eligible cohort participants were automatically enrolled into the trial after eligibility screening. Participants in intervention villages, were offered the intervention services by their CHW, including the offer of antihypertensive medication and could then accept or decline any of the offered services (oral intervention consent)."*
**8**	You will need to update the Reporting summary so that the Data and code availability statements match those provided in the manuscript	Done	**Reporting summary**<br>Updated to match data and code availability statement in the manuscript
	Please also ensure that your Data and/or Code Availability Statements comply with our requirements: https://www.nature.com/nature-portfolio/editorial-policies/reporting-standards<br>The Data and Code Availability Statements must indicate any restrictions on data/code availability. Please indicate whether individual-level data will be made available, when, and for how long. Please provide a transparent path detailing how external requests will be evaluated, including contact information and an estimated timeframe for response when applicable.	Done	**Data availability statement**<br>*"A de-identified individual-level key dataset alongside the statistical code for reproducing the primary and secondary endpoints is openly available from publication until at least 25 years thereafter at https://doi.org/10.5281/zenodo.16903262. The trial was registered with ClinicalTrials.gov (NCT05684055), where a full protocol and statistical analysis plan are available. A study protocol manuscript has been published previously[2]. Requests for access to more*

	In the code availability statement, we do not allow “data available upon reasonable request”. Please provide more details regarding how the test environment of the CDSS can be made available upon application, including timelines<br>In the Data Availability Statement- under “Requests for access to more detailed data may be made to the corresponding author by submitting a proposal, which will be reviewed by the trial consortium.”- please provide more details regarding timelines of when granting access would be approved		*detailed data may be made to the corresponding author by submitting a proposal. The proposal will be reviewed by the corresponding author and access granted if deemed reasonable within four weeks.”*<br>**Code availability statement**<br>*“The code for the CDSS application used by CHWs for data collection and service delivery is available at https://github.com/clinepi-usb/cht-combacal. Access to the test environment of the CDSS application is available from the corresponding author upon written request. The request will be reviewed by the corresponding author and credentials for the test environment shared if deemed reasonable within four weeks.”*
	When revising your manuscript:<br>- Submit both clean and tracked changes versions of the revision as editable Word documents.<br>- Include a “Response to referees and editors” document detailing, point-by-point, how you addressed each referee and editorial comment.<br>- Include a revised version of any required reporting checklist, including the Reporting Summary (https://www.nature.com/documents/nr-reporting-summary-flat.pdf). It will be available to referees (and, potentially, statisticians) to aid in their evaluation if the manuscript goes back for peer review. A revised checklist is essential for re-review of the paper.	Done	None
	When preparing your revised manuscript, please be aware of our guidance on Sex and Gender reporting). Please note that:<br>1. If the research findings apply to only one sex or gender, that must be indicated in the title and/or abstract.	Not applicable	None
	2a. For studies involving vertebrates animal and cell lines- The Reporting Summary should include whether sex was were considered in the study design.	Not applicable	None
	2b. For studies involving human research participants- The Reporting Summary should include whether sex and/or gender was considered in the study design and whether sex and/or gender of participants was determined based on self-report or assigned (and methodology used).	Already addressed in previous submission	None

	3. Data should be reported disaggregated for sex and gender where this information has been collected and consent has been obtained for reporting and sharing individual-level data; disaggregated numbers for individual experiments must be provided in the source data as appropriate whereas overall numbers may be provided in the Nature Portfolio Reporting Summary.	Already addressed in previous submission	None
	Information on the 3 points above should be included in the revised manuscript and detailed in the cover letter.	Already addressed in previous submission	None
	In addition, please note that if sex- and gender-based analyses have been performed a priori, results should be reported regardless of positive or negative outcome. We discourage conducting post hoc sex- and gender-based analysis if the study design is insufficient (for example, low sample size) to enable meaningful conclusions.	Already addressed in previous submission:<br>**Sample size and statistical analysis:**<br>"*Modification of the intervention effect on the primary endpoint was assessed for the following predefined factors: eligibility for pharmacological treatment by the CHW (excluding participants requiring three or more antihypertensive drugs or having an intolerance against amlodipine or hydrochlorothiazide), age, sex, engagement in antihypertensive care at baseline, engagement in HIV care at baseline, access to health facility. Only if the p-values of an interaction term were found to be below 0.1, formal subgroup analyses and subgroup credibility assessments are conducted.*"<br>**Results:**<br>"*Interaction p-values for the prespecified potential effect modifiers (sex, age, engagement in hypertension care at baseline, access to health facility, and engagement in*	None

		HIV care) were larger than 0.1 and therefore no subgroup credibility assessments were performed (see extended data table 4 and extended data figure 1)."	
	If no sex- and gender-based analyses have been performed, please indicate the reasons for the lack of these analyses in the Reporting Summary	Already addressed in previous submission	None
	When submitting the revised version of your manuscript, please also pay close attention to our href="https://www.nature.com/nature-research/editorial-policies/image-integrity">Digital Image Integrity Guidelines. and to the following points below:<br>-- that unprocessed scans are clearly labelled and match the gels and western blots presented in figures.<br>-- that control panels for gels and western blots are appropriately described as loading on sample processing controls<br>-- all images in the paper are checked for duplication of panels and for splicing of gel lanes.	Already addressed in previous submission	None
	Finally, please ensure that you retain unprocessed data and metadata files after publication, ideally archiving data in perpetuity, as these may be requested during the peer review and production process or after publication if any issues arise.	Data and metadata files will be archived for at least 25 years after publication.	None
